# Comparing Vegetation Characteristics and Production Outcomes of Semi-Natural Permanent Grassland and Sown Ley: A Case Study in a Hemi-Boreal Region

**DOI:** 10.3390/plants15193026

**Published:** 2026-10-02

**Authors:** Vilma Živatkauskienė, Vaclovas Stukonis, Sigitas Juzėnas, Gintarė Zaborskienė, Vilma Kemešytė, Eglė Norkevičienė

**Affiliations:** 1 Lithuanian Research Centre for Agriculture and Forestry, Instituto al. 1, Akademija, LT-58344 Kedainiai, Lithuania; vilma.zivatkauskiene@lammc.lt (V.Ž.); vaclovas.stukonis@lammc.lt (V.S.); vilma.kemesyte@lammc.lt (V.K.); 2 Life Sciences Center, Institute of Biosciences, Vilnius University, Sauletekio Av. 7, LT-10257 Vilnius, Lithuania; sigitas.juzenas@gf.vu.lt; 3 Department of Food Safety and Quality, Faculty of Veterinary Medicine, Lithuanian University of Health Sciences, LT-44307 Kaunas, Lithuania; gintare.zaborskiene@lsmu.lt

**Keywords:** plant diversity, functional homogenization, forage nutritive value, forage phytochemistry, phenolic compounds, antioxidant activity, fatty acid composition, lipid quality indices, cattle grazing, Natura 2000

## Abstract

Semi-natural grasslands support biodiversity, but their capacity to combine conservation with beef production remains insufficiently quantified. This case study compared a sown ley and a permanent semi-natural grassland in Lithuania during the 2019–2020 grazing seasons, using eight bulls annually (16 animal observations; four per field per year). Plant diversity, herbage yield and composition, liveweight change, and beef quality were evaluated. Soil phosphorus status differed, with lower extractable phosphorus in the permanent grassland. Species richness was higher in permanent grassland than in sown ley (84 vs. 12 species), whereas herbage dry matter yield was lower (1.45 vs. 2.92 t DM ha ^−1^). Permanent grassland had half the livestock unit density and twice the land area per animal, resulting in similar dry matter yield per animal. Despite the greater land requirement, unadjusted growth differences varied in direction between years, and the adjusted difference in final liveweight was imprecisely estimated (permanent grassland minus ley: −10.1 kg; 95% CI: −39.7 to 19.5 kg). Beef from the studied permanent grassland had less saturated and more unsaturated fatty acids, lower atherogenicity and thrombogenicity indices, and a higher hypocholesterolaemic-to-hypercholesterolaemic fatty acid ratio. Multi-site studies are needed to assess the broader applicability of these differences.

## 1. Introduction

Permanent grasslands account for approximately one-third of the utilised agricultural area in the European Union [1]. This broad land-use category includes grasslands that differ substantially in origin, management history, botanical composition, and conservation importance. Ploughing and agricultural intensification simplify their plant communities and reduce the ecological functions they support. At the other end of the management gradient, the withdrawal of mowing or grazing allows succession to proceed and gradually removes species associated with open vegetation [2,3]. Semi-natural grasslands therefore require continued, low-intensity mowing or grazing to maintain their characteristic open vegetation [4]. Their persistence ultimately depends on whether this management remains practical for farmers or has to be sustained through targeted conservation measures.

Agricultural declarations indicate that the role of long-term grassland in Lithuanian farming is declining. Based on the categories applied in each year, the area recorded as permanent grassland declined by approximately 29% between 2017 and 2024 [5]. Although these categories do not directly correspond to Annex I habitat classes, less land is being designated as long-term grassland.

The conservation status of Annex I grassland and pastoral habitats is also poor across Europe: 84% of assessments are unfavourable, and 45% indicate continuing deterioration [6]. In Lithuania, a comprehensive national survey conducted between 2012 and 2014 mapped 77,189 ha of grasslands and adjacent EU-protected habitats [7]. Within only four years, however, 9% of the mapped habitats had been altered or lost, mainly through conversion to arable fields, cultivated grasslands and forest plantations. This documented habitat loss is consistent with recent national assessments identifying abandonment, conversion to cultivated grassland or arable land, declining livestock numbers and the disappearance of small farms as major pressures on semi-natural grasslands in the country [8].

Sown leys are currently often preferred because they provide relatively high and more uniform biomass yields, contain relatively high levels of crude protein, and are compatible with intensive livestock systems. By contrast, semi-natural grasslands may be less productive, or produce forage of lower conventional nutritive value, particularly at later developmental stages. Herbage yield and crude-protein content capture only part of forage functionality. Forbs and legumes occurring in species-rich swards may provide minerals, phenolic acids, flavonoids, and other bioactive compounds that are not represented by conventional measures of forage yield, protein, or fibre. These compounds may contribute to forage antioxidant activity and variation in animal-product composition [9,10,11,12,13]. Greater plant diversity does not necessarily result in lower agricultural production. Biomass yield and forage quality depend on the species present and how the grassland is managed, and some diverse grasslands can achieve competitive biomass production and quality-adjusted yields [14]. Sown leys may offer clear advantages in herbage yield and predictability, but these gains can coincide with a narrower range of plant traits and forage constituents. Species-rich permanent grasslands provide a contrasting system in which lower herbage production may be accompanied by greater botanical and functional diversity, as well as differences in biomass chemistry. Whether these differences are reflected in animal performance or product composition remains less clear.

Studies often examine vegetation, forage, and animal-product characteristics separately rather than as components of an integrated pathway. Evidence from pasture-based beef systems suggests that botanical composition and forage phytochemicals may contribute to the antioxidant and fatty acid composition of beef [15,16], although the responses vary with plant composition, grazing regime and season, animal characteristics, and meat fat content [15,17,18,19,20].

In this context, the study compared a semi-natural permanent grassland within a Natura 2000 habitat site (hereafter referred to as permanent grassland) with a sown ley, together with the bulls grazing the two fields. We tested whether permanent grassland differed from sown ley not only in plant diversity, but also in functional structure, forage productivity, biochemical composition, animal performance, and beef-quality traits. Specifically, we tested the following hypotheses: H1, permanent grassland would exhibit greater taxonomic diversity and greater abundance-weighted differentiation within the persistence, regeneration, and dispersal-strategy dimensions represented by the analysed traits; H2, sown ley would produce greater biomass and show more favourable conventional forage quality indicators; H3, the permanent grassland would exhibit higher forage phenolic content and antioxidant activity; and H4, the bulls grazing the two fields would differ in growth, conventional physicochemical meat-quality traits, and beef lipid-quality characteristics. By jointly evaluating vegetation, forage, animal performance, and beef-quality traits, the study assesses how the conservation value of the studied permanent grassland field was associated with measurable agricultural and product-quality outcomes, rather than being represented by plant species richness alone.

## 2. Results

### 2.1. Taxonomic Diversity

**Figure 1:**
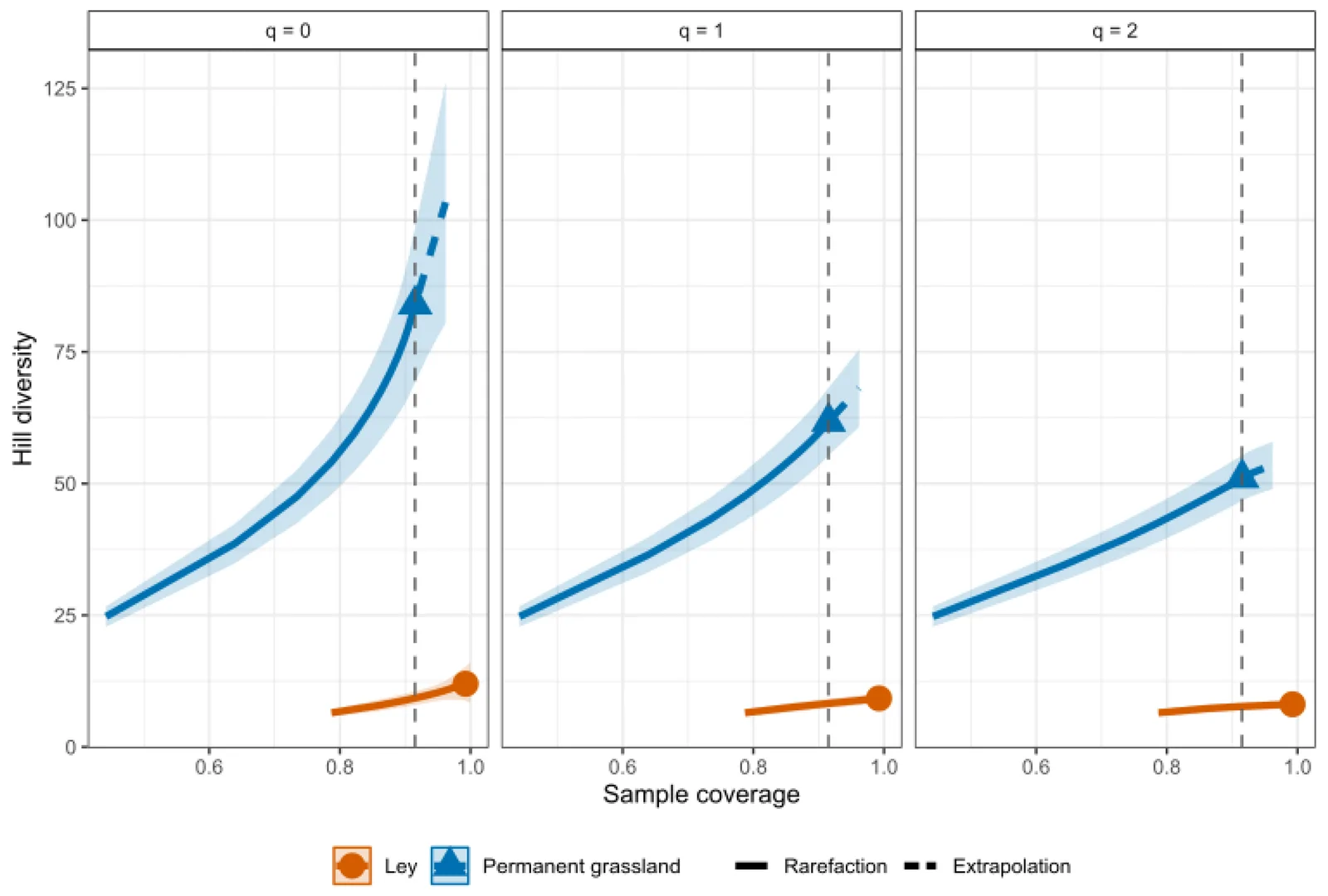
Coverage-based rarefaction and extrapolation curves for incidence-based Hill diversity in the ley and permanent grassland. Hill numbers of orders q = 0, q = 1, and q = 2 represent species richness, Shannon effective diversity, and Simpson effective diversity, respectively. Solid and dashed curve segments represent interpolation and extrapolation, respectively, and shaded areas indicate 95% bootstrap confidence intervals calculated using 9999 bootstrap replications. The vertical dashed line marks the common sample coverage of 91.50% used for the primary comparison. Diversity was calculated from species incidences across 1 m^2^ vegetation plots.

The two studied fields differed substantially in taxonomic diversity (Figure 1). The reference samples contained 84 species in the permanent grassland and 12 species in the sown ley, with six species shared between the two fields. Observed sample coverage was 99.24% in the sown ley and 91.53% in the permanent grassland. Therefore, the primary comparison was standardised to a common sample coverage of 91.50%, indicated by the vertical dashed line in Figure 1. Because this target was slightly below the observed coverage of the permanent grassland and substantially below that of the ley, both assemblages were rarefied to the same coverage. The adjustment was minimal for the permanent grassland, resulting in an estimated richness of 83.88 rather than the observed count of 84 species. At this common coverage, Hill diversity was higher in the permanent grassland than in the ley for species richness (q = 0: 83.88, 95% CI 69.17–98.59, versus 9.28, 95% CI 7.96–10.60), Shannon effective diversity (q = 1: 61.72, 95% CI 55.28–68.16, versus 8.27, 95% CI 7.35–9.18), and Simpson effective diversity (q = 2: 51.06, 95% CI 46.73–55.38, versus 7.69, 95% CI 6.93–8.45). The 95% confidence intervals did not overlap for any Hill order. However, the lower coverage and the continuing increase in the q = 0 curve for the permanent grassland indicate that additional sampling would likely have detected further infrequent taxa. Observed and asymptotic Hill-number estimates and additional sampling diagnostics are provided in Supplementary File S1.

### 2.2. Functional Diversity and Ecological Indicator Gradients

Among the functional diversity metrics based on persistence, regeneration, and dispersal traits, Rao’s quadratic entropy (RaoQ) and functional dispersion (FDis) were higher in the sampled permanent grassland plots than in the sampled ley plots (Table 1), whereas functional evenness (FEve) did not differ between the two fields.

FDis values in the ley were heterogeneous, with several plots showing values close to zero. None of the 16 ley plots had an FDis value of zero, whereas five plots (31.3%) had values below 0.01. No permanent grassland plot had an FDis value of zero or below 0.01. Complete plot-level functional diversity values are provided in Supplementary File S2.

These metrics describe differences in community assembly within the ecological-strategy dimensions represented by the analysed traits and are not interpreted as mechanistic indicators of forage chemistry, nutritive value, animal performance, or beef composition. Additional functional-diversity diagnostics are provided in Supplementary File S2.

**Figure 2:**
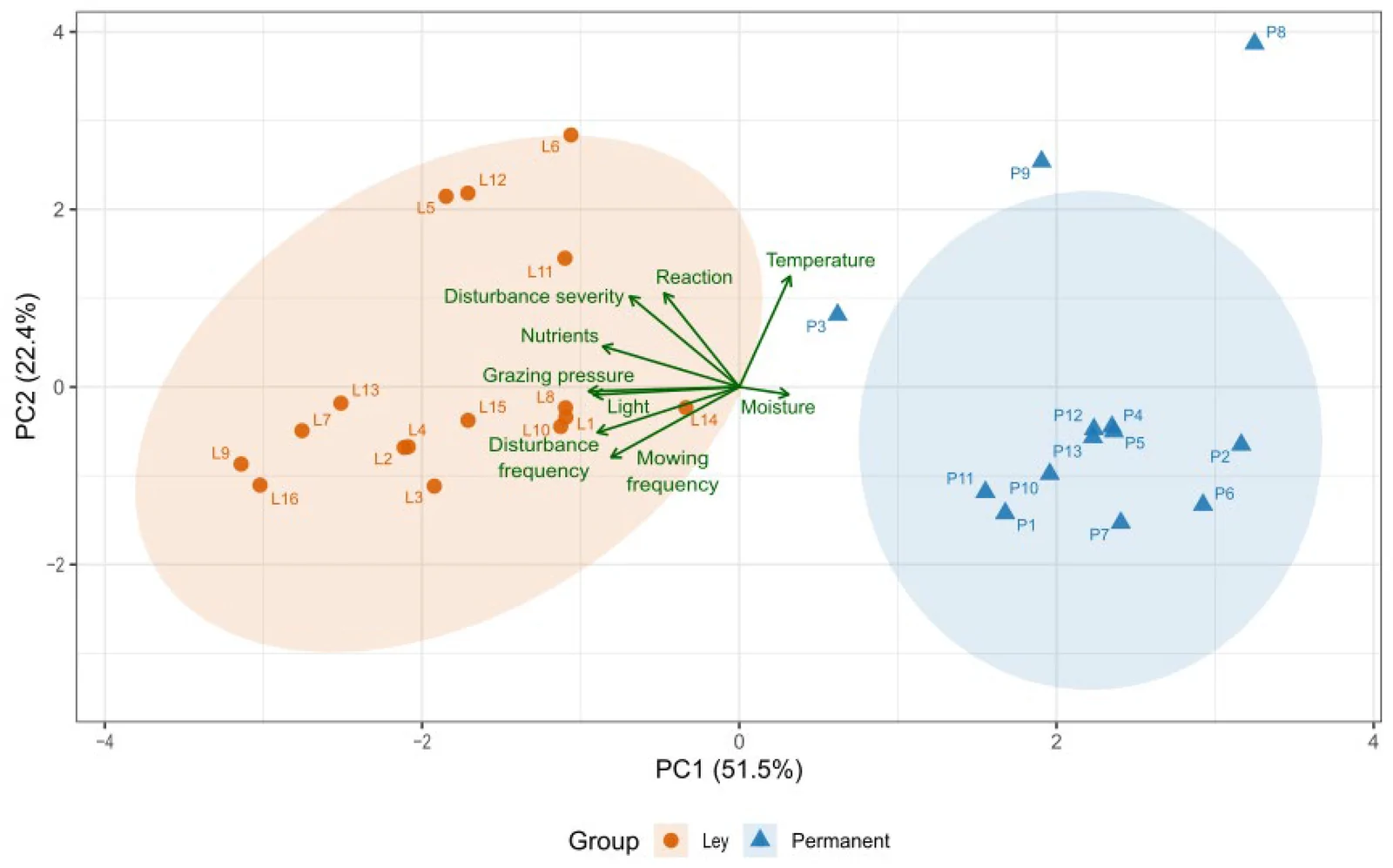
Principal component analysis of plot-level community-weighted mean ecological indicator values in the studied ley and permanent grassland fields. The shaded orange and blue areas represent the 95% t-distribution ellipses for ley and permanent grassland plots, respectively. Arrows represent indicator loadings.

The PCA of plot-level community-weighted mean ecological indicator profiles showed clear differentiation between the two studied fields (Figure 2). These profiles represent the ecological affinities of the constituent plant species rather than direct measurements of environmental conditions or management intensity.

The first two principal components explained 73.96% of the variation in community-weighted ecological indicator values. The two fields were separated mainly along PC1, whereas PC2 captured additional variation among plots. Ley plots occurred in the direction of higher indicator values for disturbance frequency and severity, mowing frequency, grazing pressure, light, and nutrients. Permanent grassland plots were positioned on the opposite side of the ordination and had lower community-weighted values for most management related indicators.

PERMANOVA based on the full nine-indicator profile indicated significant separation between plots from the two studied fields (R^2^ = 0.459, F = 22.90, *p* < 0.001). This separation was not attributable to a significant difference in multivariate dispersion (PERMDISP: F = 2.00, *p* = 0.171). A robustness analysis using only high-coverage indicators showed a similar pattern of separation (R^2^ = 0.528, F = 30.21, *p* < 0.001). Therefore, the observed separation between the studied fields was retained when the lower-coverage temperature, moisture and nutrient indicators were excluded. These lower-coverage indicators were interpreted only as exploratory gradients.

### 2.3. Biomass Productivity and Chemical Composition

Biomass yield and chemical characteristics, together with outcome-specific tests and FDR-adjusted results, are presented in Table 2.

Mean dry matter yield (DMY) was 2.92 ± 1.56 t ha^−1^ in the ley and 1.45 ± 0.86 t ha^−1^ in the permanent grassland, and the difference remained statistically significant after FDR correction (q = 0.0165). Although mean fresh matter yield (FMY) was numerically higher in the ley, this contrast did not remain statistically significant after correction (q = 0.0613). The permanent grassland biomass had higher acid detergent fibre (ADF) and neutral detergent fibre (NDF) concentrations estimated by NIRS, as well as higher total phenolic content (TPC) and DPPH radical-scavenging activity. All four differences remained statistically significant after FDR correction. NIRS-estimated CP and CF did not differ significantly between fields (Table 2).

Botanical-group composition differed between the sampled quadrats of the two fields (PERMANOVA: pseudo-F = 64.36, R^2^ = 0.704, *p* < 0.001). However, PERMDISP indicated unequal multivariate dispersion (F = 5.03, *p* = 0.032), with greater dispersion in the permanent grassland. Therefore, the PERMANOVA result may reflect both differences in botanical-group composition and greater within-field heterogeneity in the permanent grassland. The ley was dominated by grasses and legumes, whereas the permanent grassland had a more heterogeneous composition, characterised by a substantially greater proportion of forbs and a patchy occurrence of sedges (Table 3).

**Figure 3:**
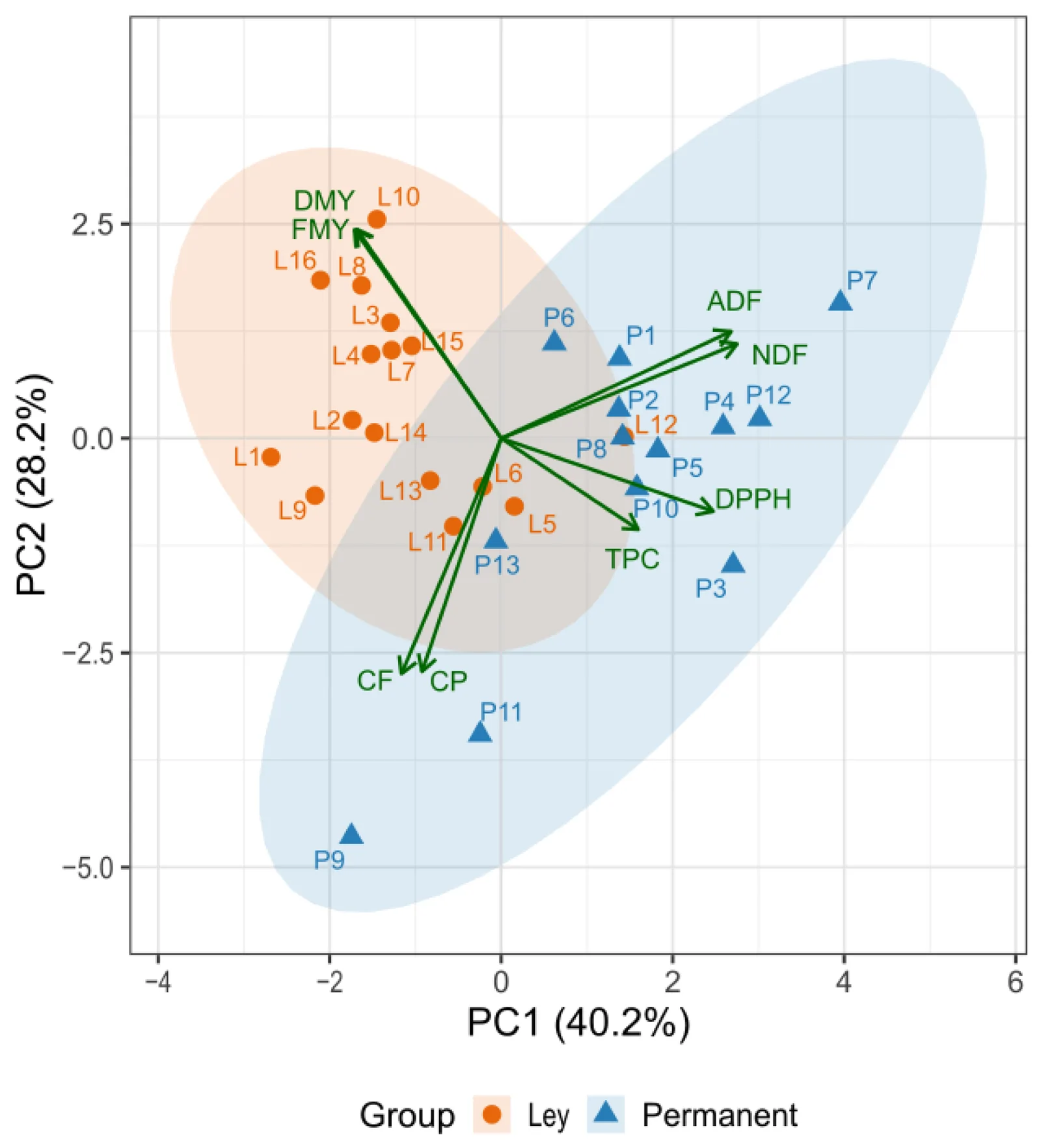
Principal component analysis of plot-level biomass yield and chemical composition in the studied ley and permanent grassland fields. The shaded orange and blue areas represent the 95% normal-probability ellipses for ley and permanent grassland plots, respectively. Arrows represent variable loadings: FMY, fresh matter yield; DMY, dry matter yield; ADF, acid detergent fibre; NDF, neutral detergent fibre; CP, crude protein; CF, crude fat; TPC, total phenolic content; and DPPH, DPPH radical-scavenging activity.

The PCA summarised a substantial portion of the variation in the combined biomass-yield and chemical-composition dataset. PC1 explained 40.2% of the total variation, and PC2 explained 28.2%; together, the first two components accounted for 68.4% of the variation in the standardised dataset (Figure 3).

PC1 represented the main contrast between fibre- and antioxidant-related variables and biomass yield. The strongest positive loadings on PC1 were observed for NDF (0.491), ADF (0.478) and DPPH (0.441), followed by TPC (0.285). In contrast, FMY (−0.306) and DMY (−0.301) loaded negatively on PC1, with weaker negative loadings for CF (−0.208) and CP (−0.165). Thus, positive PC1 scores were associated mainly with higher NDF, ADF, DPPH and TPC values, whereas negative PC1 scores were associated more closely with higher FMY and DMY.

PC2 primarily separated yield-related variation from crude protein and crude fat concentrations. DMY (0.435) and FMY (0.434) had the strongest positive PC2 loadings, while CF (−0.490) and CP (−0.485) had the strongest negative PC2 loadings. ADF (0.222) and NDF (0.196) contributed moderately to the positive side of PC2, whereas TPC (−0.190) and DPPH (−0.152) contributed weakly to the negative side. Therefore, PC2 mainly described a contrast between yield-related variables and crude protein and crude fat concentrations.

The combined biomass-yield and chemical-composition profile differed between ley and permanent grassland plots according to PERMANOVA (F = 9.21, R^2^ = 0.254, *p* < 0.001). PERMDISP indicated no significant difference in multivariate dispersion (F = 3.87, *p* = 0.0565), suggesting that the PERMANOVA result was mainly attributable to differences in multivariate group location rather than to a statistically confirmed difference in dispersion. Nevertheless, because the PERMDISP result was close to the significance threshold, the apparently wider spread of permanent grassland samples in the PCA plot was interpreted cautiously.

### 2.4. Animal Growth Performance

Initial liveweight showed no statistically supported difference between the two animal groups within either year. Mean initial liveweights in the ley and permanent grassland groups were 435.3 ± 84.8 and 440.5 ± 72.9 kg in 2019 and 446.3 ± 32.7 and 431.0 ± 47.1 kg in 2020, respectively. Exact permutation tests provided no evidence of a between-group difference in either year (*p* = 0.943 and *p* = 0.657).

The direction of the unadjusted difference in total liveweight change varied between years. In 2019, mean total change was 165.0 ± 49.4 kg in the ley group and 139.3 ± 65.0 kg in the permanent grassland group. In 2020, the corresponding values were 135.5 ± 36.3 and 147.5 ± 35.2 kg, respectively. Average daily liveweight change was calculated over the verified 130-day interval between weighing occasions, comprising the 30-day adaptation phase and the subsequent 100-day grazing phase. The mean values were 1.27 and 1.07 kg d^−1^ in the ley and permanent grassland groups, respectively, in 2019, and 1.04 and 1.13 kg d^−1^, respectively, in 2020 (Table 4).

Carcass characteristics by grassland group and year are presented descriptively in Table 4 and were not subjected to separate inferential comparisons because each field × year combination included only four animals. The predominance of EUROP fat class 2 was consistent with the relatively low intramuscular fat concentrations observed across the study groups.

Final liveweight was analysed using ANCOVA, with initial liveweight as a covariate and studied field and year as fixed terms. Initial liveweight was positively associated with final liveweight: each 1 kg increase in initial liveweight was associated with an estimated 0.349 kg increase in final liveweight (95% CI: 0.078 to 0.620; F = 7.90; Freedman–Lane permutation *p* = 0.016). The small standard deviation of final liveweight in the 2020 ley group reflects the four verified individual records, which ranged from 574 to 586 kg, and was not caused by a calculation or data-grouping error.

After adjustment for initial liveweight and year, the estimated marginal mean final liveweight was 590.1 kg (95% CI: 569.2 to 611.0) in the ley group and 580.0 kg (95% CI: 559.1 to 600.9) in the permanent grassland group. The adjusted permanent grassland minus ley contrast was −10.13 kg (95% CI: −39.73 to 19.47; F = 0.56; permutation *p* = 0.472).

No statistically supported year effect was detected. In the exploratory interaction model, the adjusted permanent grassland minus ley contrast was −22.39 kg in 2019 (95% CI: −65.01 to 20.24) and 2.23 kg in 2020 (95% CI: −40.57 to 45.04). The interaction coefficient was 24.62 kg (95% CI: −35.9 to 85.1), and the overall studied field × year interaction was not statistically supported (F = 0.80; permutation *p* = 0.388). Because only four animals were included in each field × year combination, the interaction result was interpreted cautiously.

### 2.5. Physicochemical Properties and Proximate Composition of Meat

After FDR correction within the physicochemical-property and proximate-composition outcome families, no significant group effect was detected for dry matter, pH, colour parameters, drip loss, water-holding capacity, cooking loss, intramuscular fat (IMF), ash, or protein content (Table 5).

After FDR correction, significant year effects were retained for IMF content and meat redness (a*), both of which were higher in 2020 than in 2019 (q = 0.002 and q = 0.035, respectively). Yellowness (b*) differed between years in the unadjusted permutation analysis (*p* = 0.026), but not after FDR correction (q = 0.091).

No group × year interaction was significant after FDR correction. The weak indication of an interaction for cooking loss (*p* = 0.066) was not supported after correction within the seven-outcome physicochemical-property family (q = 0.462). The apparent reversal in group means between years was therefore not considered evidence of year-dependent group differences in these traits.

### 2.6. Fatty Acid Profile and Lipid Quality Indices

Exploratory animal-level comparisons showed differences in relative intramuscular fatty acid composition between the two grazing groups. After adjustment for year and multiple testing, the model-adjusted SFA proportion was lower in the permanent grassland group than in the ley group: 44.46% (95% CI: 43.90–45.03) versus 50.19% (95% CI: 49.63–50.75). The permanent minus ley difference was −5.73 percentage points (95% CI: −6.53 to −4.90; q < 0.001).

**Figure 4:**
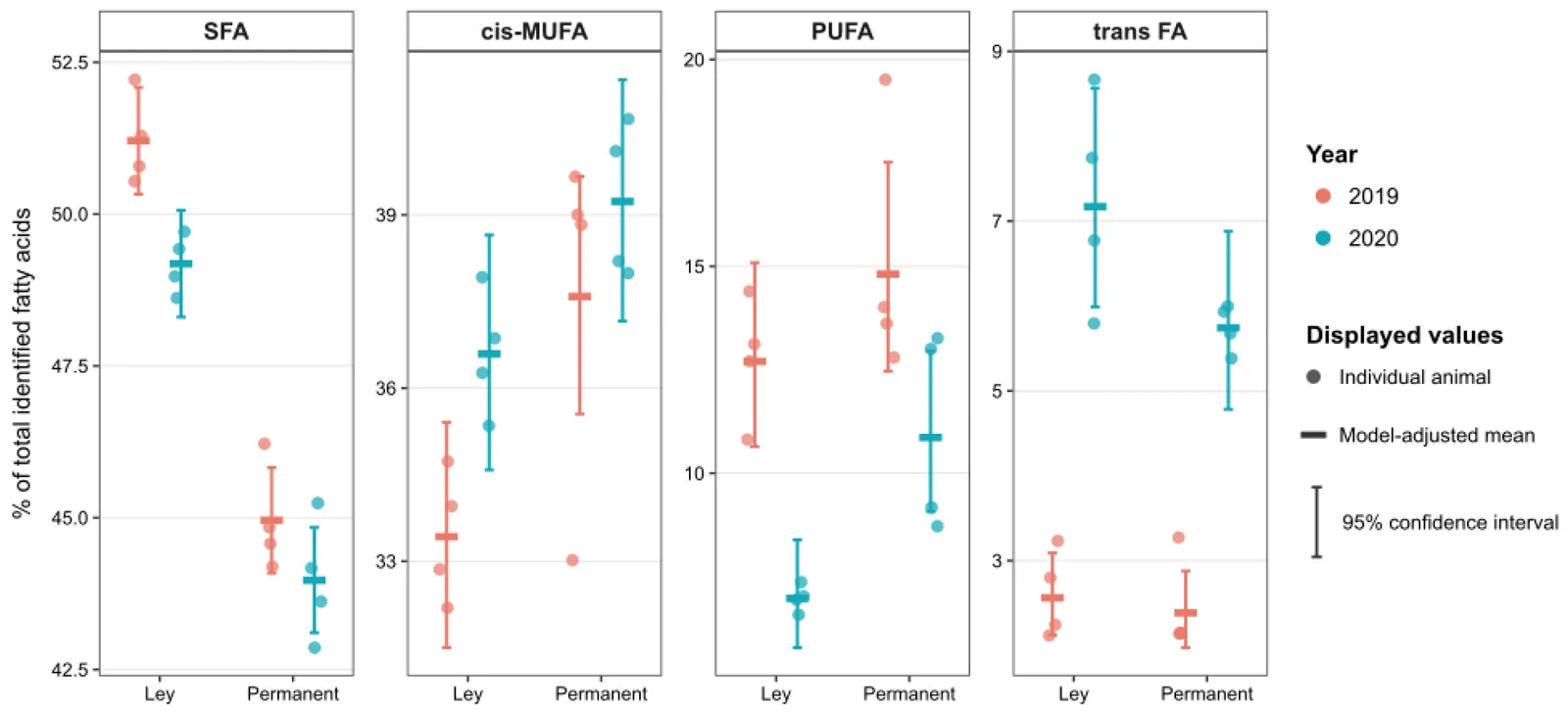
Intramuscular fatty acid class composition in young bulls from the ley and permanent grassland groups in 2019 and 2020. Small, coloured points represent individual animals (*n* = 4 per grassland group × year combination). Horizontal lines indicate model-adjusted estimates for each grassland group × year combination, and vertical error bars indicate their 95% confidence intervals. Colours distinguish sampling years. Individual observations were horizontally dispersed to reduce overlap. Models included grassland group, year, and their interaction. Estimates shown in the figure are specific to each grassland group × year combination, whereas Table 6 reports marginal grassland-group estimates equally weighted across 2019 and 2020. FDR-adjusted tests of the overall grassland group effect are reported in Table 6.

By contrast, the model-adjusted cis-MUFA and PUFA proportions were higher in the permanent grassland group. cis-MUFA accounted for 38.41% (95% CI: 37.07–39.76) in the permanent grassland group and 35.01% (95% CI: 33.73–36.29) in the ley group, corresponding to a difference of 3.40 percentage points (95% CI: 1.54–5.34; q = 0.006). The respective PUFA proportions were 12.84% (95% CI: 11.46–14.38) and 9.84% (95% CI: 8.75–11.06), with a difference of 3.00 percentage points (95% CI: 1.12–4.95; q = 0.006) (Figure 4; Table 6). Because the fatty acid class proportions were compositionally constrained, the lower relative SFA proportion and the higher relative cis-MUFA and PUFA proportions represent components of the same compositional shift rather than statistically independent changes.

Year effects remained significant for SFA, cis-MUFA and PUFA after FDR correction, indicating differences between the 2019 and 2020 animal cohorts. None of these endpoints showed a significant group × year interaction, providing no evidence that the contrasts between grazing groups differed between years.

The analytically unresolved trans FA class differed between years (q < 0.001) but not between grassland groups after FDR correction (q = 0.104). The model-adjusted Permanent minus Ley difference was −0.80 percentage points (95% CI: −1.63 to 0.03). Because individual trans-C18:1 positional isomers could not be resolved and verified retrospectively, this aggregate outcome was treated as exploratory and was not used to support the principal biological interpretation of the fatty acid results.

The higher total PUFA proportion in the permanent grassland group was accompanied by higher model-adjusted proportions of both n-3 and n-6 PUFA. The n-3 PUFA proportion was 3.98% (95% CI: 3.22–4.96) in the permanent grassland group and 2.69% (95% CI: 2.15–3.41) in the ley group, corresponding to a difference of 1.29 percentage points (95% CI: 0.25–2.43; q = 0.007). The respective n-6 PUFA proportions were 8.81% (95% CI: 7.77–10.01) and 7.07% (95% CI: 6.21–8.04), with a difference of 1.75 percentage points (95% CI: 0.30–3.26; q = 0.027). Year effects remained significant for both endpoints after FDR correction, whereas neither showed a significant group × year interaction.

The model-adjusted PUFA/SFA ratio was also higher in the permanent grassland group than in the ley group: 0.293 (95% CI: 0.254–0.332) versus 0.196 (95% CI: 0.157–0.235), corresponding to a difference of 0.097 (95% CI: 0.042–0.153; q = 0.005). The year effect remained significant after FDR correction.

The log-transformed analysis of the n-6/n-3 ratio did not support a difference between the grassland groups. The permanent/ley ratio of geometric marginal means was 0.756 (95% CI: 0.528–1.082; q = 0.115; Table 6). The n-6/n-3 ratio differed between years (q = 0.010), but there was no significant group × year interaction (q = 0.327) (Table 6). Taken together, these results indicate a broader shift in relative PUFA composition, involving higher relative proportions of both n-3 and n-6 PUFA, rather than selective n-3 enrichment.

The lipid quality indices differed between groups. The model-adjusted atherogenicity index (AI) was lower in the permanent grassland group than in the ley group: 0.700 (95% CI 0.648–0.752) versus 0.906 (95% CI 0.854–0.958), respectively. The adjusted difference was −0.206 (95% CI −0.280 to −0.132; q < 0.001).

Similarly, the model-adjusted thrombogenicity index (TI) was lower in the permanent grassland group than in the ley group: 1.096 (95% CI: 1.034–1.159) versus 1.535 (95% CI: 1.473–1.598), corresponding to a difference of −0.439 (95% CI: −0.528 to −0.350; q < 0.001).

In contrast, the adjusted hypocholesterolaemic/hypercholesterolaemic fatty acid ratio (h/H) ratio was higher in the permanent grassland group than in the ley group: 1.591 (95% CI 1.534–1.647) versus 1.258 (95% CI 1.201–1.314), respectively. The adjusted difference was 0.333 (95% CI 0.253–0.413; q < 0.001) (Table 6).

Year effects remained significant after FDR correction for AI and TI, whereas h/H did not differ between years (q = 0.820). No group × year interaction remained significant after FDR correction for AI, TI or h/H. In particular, the unadjusted TI interaction *p*-value of 0.057 corresponded to q = 0.313 and was not interpreted as evidence that the contrast between groups differed between years.

Descriptive statistics for the reliably identified individual fatty acids by grassland group and year are provided in Supplementary File S3. Fatty acids that could not be resolved or verified separately are identified accordingly. Adjustment for individual IMF content did not materially alter the estimated grassland-group contrasts. For SFA, the permanent grassland minus ley contrast was −5.73 percentage points (95% CI −6.53 to −4.90) in the base model and −5.82 percentage points (95% CI −6.65 to −4.99) after adjustment for IMF. The complete base and IMF-adjusted results are reported in Supplementary File S4.

Overall, under the conditions of the present study, IMF from bulls in the permanent grassland group was characterised by a lower relative proportion of SFA, higher proportions of cis-MUFA, PUFA, n-3 PUFA and n-6 PUFA, a higher PUFA/SFA ratio, lower AI and TI values, and a higher h/H ratio than IMF from bulls in the ley group. These animal-level contrasts remained statistically significant after Benjamini–Hochberg FDR correction across the 11 meat lipid outcomes. The n-6/n-3 ratio did not differ significantly between groups after FDR correction, while the analytically unresolved trans FA class was treated as exploratory.

## 3. Discussion

The taxonomic contrast between the two sampled fields extended beyond the number of species recorded. Diversity remained substantially greater in the permanent grassland when more weight was assigned to taxa occurring repeatedly across the sampled plots, indicating that the contrast was not driven solely by species restricted to a few plots. Standardisation at a common sample coverage of 91.50% supported the comparison, although the still-rising richness curve indicates that additional sampling would probably have detected further infrequently occurring species in the permanent grassland.

The permanent grassland plots also had higher cover-weighted RaoQ and FDis than the ley plots. Functional diversity complements species richness by describing variation in the ecological traits represented within a community [21]. The fields therefore differed not only in species richness but also in the abundance-weighted differentiation and dispersion of the analysed life-form, seed-mass, and dispersal-trait combinations. The recording of only 12 plant species in the ley, compared with the 11 species originally sown, further indicates the limited compositional differentiation of this sward and is consistent with its comparatively homogeneous taxonomic and functional structure.

By contrast, FEve did not differ detectably between fields. The functional contrast therefore concerned trait differentiation and dispersion rather than the evenness of trait representation. Such divergence among functional diversity indices is expected because they describe complementary components of community structure [22]. The selected traits reflect persistence, regeneration, and dispersal strategies [23], but do not directly represent resource acquisition, digestibility, tissue nutrient composition, or secondary metabolism. Functional diversity and biomass chemistry were consequently interpreted as separate characteristics of the same two fields, without assuming a mechanistic relationship between them.

The two fields also differed markedly in soil reaction and extractable nutrient status. Because the soil measurements were based on two composite samples per field, these values are descriptive and do not support inferential comparisons between fields. The sown ley had a near-neutral soil reaction and substantially higher extractable P_2_O_5_ and K_2_O concentrations than the permanent grassland, whereas the permanent grassland soil was moderately acidic and had much lower extractable phosphorus. Low available soil phosphorus may have contributed to the greater plant species richness of the permanent grassland, as plant species richness has been shown to be consistently negatively related to soil phosphorus across European grasslands [24]. These site-specific soil differences may have contributed to the observed contrasts in herbage productivity, botanical composition, and plant diversity. However, their influence cannot be separated from differences in management history, sward origin, and species composition in this single-field comparison. Descriptively, soil organic carbon was higher in the permanent grassland than in the ley (1.50% vs. 1.32%), although this numerical difference was considerably smaller than the contrasts in soil reaction and extractable phosphorus. These relationships were not tested experimentally and should not be interpreted as independent causal effects. Rather, they reinforce that grassland category was confounded with field-specific soil fertility, management history, and sward origin in this case study.

The community-weighted nutrient indicator was consistent with the higher measured extractable P and K concentrations in the ley soil. By contrast, the community-weighted reaction indicator did not follow the measured soil pH difference. This discrepancy illustrates that ecological indicator values describe the affinities of the constituent species and should not be treated as direct substitutes for measured soil properties.

Differences between the two fields were also evident in the harvested biomass profile. The higher ADF and NDF concentrations in the permanent grassland biomass should be interpreted in relation to sward structure, botanical composition, plant maturity, and management history rather than simply as indicating lower forage quality. Higher NDF may constrain voluntary intake through rumen fill, while higher ADF is commonly associated with lower digestibility [25]. However, structural fibre is also essential for chewing activity, rumen function, and stable fermentation. The observed fibre contrast may therefore represent a management trade-off, potentially modifiable through grazing timing or targeted defoliation [26]. The observed ADF and NDF contrasts exceeded the reported calibration SECV by 5.4- and 6.0-fold, respectively, indicating that random prediction error alone is unlikely to explain these differences. Nevertheless, their interpretation remains subject to the possibility of matrix-related systematic bias, as described in Section 4.5.1.

Permanent grassland biomass also had higher TPC and DPPH radical-scavenging activity, indicating a biochemical difference not captured by conventional fibre and protein measurements. Botanical composition may influence forage phenolic and antioxidant profiles [27,28], and plant-derived compounds may have antioxidant and antimicrobial functions [29]. However, the present study did not assess individual animal intake or directly link forage antioxidant characteristics to meat oxidative stability. These results therefore identify potentially relevant biochemical attributes of the permanent grassland biomass rather than demonstrating their transfer to animal products. More broadly, the findings illustrate the value of assessing permanent grasslands using botanical composition, functional-strategy diversity, and biochemical characteristics alongside biomass yield and conventional forage-quality indicators [14,30].

The direction of the unadjusted total liveweight difference varied between years. The adjusted permanent grassland minus ley contrast in final liveweight was −10.13 kg, with a wide 95% confidence interval of −39.73 to 19.47 kg. The ANCOVA coefficient for initial liveweight was 0.349, with a 95% confidence interval from 0.078 to 0.620, and was therefore below unity. This attenuated relationship may reflect convergence in final liveweight, compensatory growth, regression to the mean, and uncertainty arising from single unfasted weighings; however, the small sample size does not allow these explanations to be distinguished. Overall, the analysis provided no statistically supported evidence of a group difference, but its limited precision precludes a conclusion of equivalent growth performance. Cold carcass weight, which is less directly affected by variation in gastrointestinal fill than final liveweight, was descriptively higher for bulls grazing the ley in both years, by 22.5 kg in 2019 and 14.6 kg in 2020. These differences are reported descriptively and should not be interpreted as evidence of a grassland-group effect.

The animal-level comparison must also be interpreted in relation to the contrasting stocking rates and the limited characterisation of seasonal forage supply. Although the bulls remained in their assigned study fields throughout the complete 130-day interval between weighing occasions, they were additionally supplemented with hay during the initial 30-day phase. The herbage measurements and nominal standing DM calculations, however, related specifically to the subsequent 100-day monitored grazing phase. Therefore, the average daily liveweight changes can be interpreted in relation to the assigned fields over the complete interval, but not as performance attributable exclusively to grazing during the 100-day monitored phase or as a direct response to the single mid-June measurement of standing herbage DM.

Animal performance in forage-based systems using semi-natural pastures depends on the broader production system, including indoor feeding intensity, the number of grazing seasons, and slaughter age [31,32]. Grazing regime and vegetation selection may also influence liveweight gain and the use of heterogeneous semi-natural pasture resources [33]. The approximately twofold lower mid-June DM yield of the permanent grassland was accompanied by an approximately twofold lower stocking rate, whether expressed in LU ha^−1^ or kg liveweight ha^−1^, resulting in a similar arithmetic baseline standing crop per bull but requiring about twice as much land per animal. However, this single standing-crop measurement cannot account for forage consumed over the full grazing season. Herbage regrowth, residual biomass, grazing losses, sward height, diet selection, and individual DM intake were not measured, and the study therefore does not provide a complete seasonal forage or metabolisable-energy balance.

Average daily liveweight changes were calculated from a single initial and a single final liveweight measurement for each animal, without measurement of gastrointestinal fill or adjustment for shrink. Accordingly, these values should be interpreted as liveweight changes over the interval between weighing occasions rather than as precise estimates of tissue gain. The absence of a statistically supported difference in adjusted final liveweight therefore does not imply comparable animal production per unit area. The greater land requirement of the permanent grassland should be considered alongside its biodiversity-conservation value and the role of agri-environmental support in maintaining semi-natural grasslands [34].

After multiple-testing correction, no statistically significant grassland-group effects were detected for the physicochemical or proximate-composition outcomes. However, the small number of animals limited the ability to detect moderate differences, and these non-significant results should not be interpreted as evidence of equivalent conventional meat-quality characteristics between the groups. Similar findings have been reported in grassland-based beef systems, where cooking loss and water-holding capacity were often less responsive to production conditions than fatness, colour, or fatty acid composition [35].

Year-related variation was more apparent than grassland-group differences for several conventional meat-quality traits. After FDR correction, IMF and redness differed between the 2019 and 2020 animal cohorts, whereas the initial difference in yellowness was no longer statistically significant. The higher IMF content in 2020 may reflect cohort differences in forage conditions, maturity, or growth [35,36]. Although the same colourimeter, settings, and calibration procedure were used, changes in instrument performance between measurement occasions cannot be excluded. In addition, colour was measured using a 2° standard observer. Although this setting cannot explain the difference between years because it was used consistently, observer angle can affect instrumental colour coordinates. The use of a 2° rather than a 10° observer may therefore be one factor contributing to the relatively high absolute a* values recorded in 2020 and should be considered when comparing these values with studies using a D65/10° configuration. The colour differences were therefore interpreted cautiously and were not assigned a specific biological mechanism.

The clearest animal-product contrast concerned relative intramuscular fatty acid composition. After adjustment for year and FDR correction across the 11 prespecified meat lipid outcomes, the permanent grassland group had a lower relative SFA proportion and higher relative cis-MUFA, PUFA, n-3 PUFA, and n-6 PUFA proportions. The PUFA/SFA ratio was also higher, indicating a shift towards a more unsaturated relative fatty acid composition. This pattern is consistent with evidence that botanically diverse grazing systems may influence nutritional and phytochemical characteristics more clearly than conventional physicochemical meat-quality traits [37,38].

These fatty acid results require a compositional interpretation. Because fatty acids were expressed as percentages of total identified fatty acids, the lower SFA and higher cis-MUFA and PUFA proportions represent interdependent components of the same relative compositional shift rather than statistically independent changes in absolute concentrations. The outcome-specific models respected the bounded scale of individual proportions but did not constitute a joint log-ratio analysis of the complete fatty acid composition. In addition, the aggregate trans FA result was not assigned a specific biochemical interpretation because individual trans-C18:1 positional isomers, including vaccenic acid, could not be resolved and quantified separately. The magnitude of the unresolved trans-C18:1 fraction was consistent with the possibility that vaccenic acid contributed substantially to this fraction; however, the available analytical information did not permit isomer-specific identification or quantification. Similarly, because rumenic acid (CLA cis-9,trans-11) was not separately verified or quantified, a possible unresolved contribution to another chromatographic component, and consequently to the reported PUFA or n-6 PUFA totals, could not be retrospectively excluded.

Adjustment for individual IMF content did not materially alter the principal group contrasts, suggesting that they were not explained solely by variation in IMF. Nevertheless, the higher IMF content in 2020 may have contributed to some of the year-related variation through dilution of phospholipid-associated PUFA by an increased triacylglycerol fraction. IMF-related dilution may therefore partly explain cohort variation but did not account for the principal contrasts between animals grazing the two fields.

Although pre-experimental feeding management was common to all animals, the fatty acid composition of the pre-experimental feeds and individual feed intake were not determined. Residual effects of the pre-experimental diet on intramuscular fatty acid composition therefore cannot be excluded. The fatty acid profiles measured at slaughter may reflect both pre-experimental nutrition and dietary exposure during the 130-day study period. Accordingly, the observed fatty acid contrasts cannot be attributed exclusively to forage consumed during the 100-day monitored grazing phase, although the bulls grazed their assigned study fields during the preceding 30-day adaptation phase.

The reported PUFA pattern indicated a broader compositional change rather than selective n-3 enrichment. The higher relative PUFA proportion in the permanent grassland group was accompanied by higher relative proportions of both n-3 and n-6 PUFA, while the n-6/n-3 ratio did not differ significantly between groups after FDR correction. Botanically diverse diets have similarly been associated with changes in linolenic acid, linoleic acid, total PUFA, and derived fatty acid ratios [15]. Differences in botanical composition, plant maturity, grazing management, and regrowth stage may alter the supply of fatty acid precursors entering ruminal biohydrogenation and subsequent tissue deposition [39]. However, the relationship between botanical composition and meat fatty acid profiles was not tested directly in the present study. The observed fatty acid contrasts cannot be attributed uniquely to botanical diversity or to permanent grassland as a general land-use category. Botanical composition, soil properties, grazing management, field history, forage availability, and other field-specific factors could not be separated from grassland category in this two-field comparison.

The lipid-quality indices were consistent with the class-level fatty acid results. AI and TI were lower, whereas h/H was higher, in the permanent grassland group. These indices summarise selected characteristics of relative fatty acid composition by assigning different weights to fatty acids with proposed atherogenic or thrombogenic properties [40]. However, they are calculated compositional descriptors rather than direct measures of clinical outcomes. The findings therefore indicate a more favourable relative lipid-quality index profile but do not demonstrate a reduction in human cardiovascular risk.

Interpretation is constrained by the comparative case-study design because each grassland category was represented by a single field. Consequently, grassland category was completely confounded with field identity, and the observed contrasts cannot be attributed uniquely to permanent grassland or sown ley as general land-use categories. The quadrat-level and animal-level observations characterised variation within the two studied fields but did not provide independent field-level replication. In addition, the small number of animals in each grassland group × year combination limited the ability to detect moderate differences in growth, carcass characteristics, and conventional meat-quality outcomes. Grassland category was also confounded with field-specific differences in soil reaction and nutrient status, particularly the marked contrast in extractable phosphorus, as well as with differences in management history and sward origin.

The single mid-June herbage sampling characterised standing herbage only at the time of sampling and did not capture seasonal changes in forage availability and composition or the diet selected by grazing animals throughout the grazing period. Because the sampling quadrats were not protected from grazing, the vegetation, biomass-yield and chemical-composition measurements represent sward conditions after approximately 30 days of grazing rather than ungrazed baseline conditions. The observed contrasts may therefore partly reflect the different grazing pressures between fields, in addition to differences in sward origin, botanical composition, soil conditions, and management history. Consequently, relationships between the measured plant-community and herbage characteristics and the animal-product outcomes remain associative. Future studies should combine repeated seasonal herbage sampling with direct or indirect assessment of animal diet selection and intake. Unequal multivariate dispersion also indicated that the botanical separation may partly reflect differences in within-field heterogeneity. FDR correction addressed multiple testing but could not overcome these design and sampling limitations.

Overall, the studied permanent grassland field combined greater taxonomic and functional plant diversity with lower herbage productivity and a distinct biomass chemical profile than the studied sown-ley field. No statistically supported differences between the animal groups were detected in growth or conventional meat-quality characteristics, although the small animal sample limited the ability to detect moderate differences. The principal animal-product contrast was a shift towards a more unsaturated relative intramuscular fatty acid composition and associated lipid-quality indices in bulls grazing the studied permanent grassland field. These findings are specific to the two studied fields and should not be interpreted as general effects of permanent grassland relative to sown ley. Confirmation requires independently replicated fields, larger animal groups, and repeated seasonal measurements of herbage availability, composition, diet selection, and animal intake.

## 4. Materials and Methods

### 4.1. Study Area and Experimental Design

The study was conducted in the Nevėžis River valley, central Lithuania (55°22′59.04″ N, 24°01′19.50″ E; WGS 84; 32 m a.s.l.), in 2019–2020. The study area lies within the Hemi-Boreal biogeographical zone and has a humid continental climate with warm summers (Köppen–Geiger class Dfb) [41]. According to the 1991–2020 standard climate normals provided by the Lithuanian Hydrometeorological Service, the long-term mean annual air temperature and annual precipitation at the nearest meteorological station were 7.9 °C and 566 mm, respectively [42]. Meteorological data for the study years were obtained from the Dotnuva meteorological station of the Lithuanian Hydrometeorological Service. During the grazing period from 17 June to 24 September 2019, the mean air temperature was 17.0 °C, and the cumulative precipitation was 187.2 mm [43]. Compared with the 1991–2020 mean for the same calendar period, the 2019 grazing period was 0.6 °C warmer and received 62.8 mm (25.12%) less precipitation. A similar pattern was observed in 2020, when the grazing period from 17 June to 24 September had a mean air temperature of 17.2 °C and cumulative precipitation of 222.1 mm. This period was 0.8 °C warmer than the 1991–2020 mean, while precipitation was 27.9 mm, or 11.16%, lower than the long-term average.

This study was designed as a comparative case study of a sown ley and a permanent grassland, each represented by a single field. Consequently, grassland type was confounded with field identity, and the observed differences cannot be attributed unequivocally to grassland type alone.

The two fields were situated on the upper part of the Nevėžis floodplain landscape. Soil context was derived from the Lithuanian national soil spatial dataset Dirv_DR10LT [44], rather than from field soil-profile validation. According to this dataset, both fields are mapped as alluvial soils in the Lithuanian national soil classification. This mapped soil unit was interpreted here as broadly corresponding to the Fluvisol reference group and indicates a sandy loam surface layer overlying sand (national map code ADb-g4/ps/s). Soil samples were collected before the beginning of the 2019 growing season. Each field was represented by two composite soil samples collected in a zigzag pattern from the 0–20 cm soil layer. The values reported below are the means of the two composite samples collected from each field. The sown-ley field had a near-neutral soil reaction (pH_KCl 7.3) and extractable P_2_O_5_ and K_2_O concentrations of 179 and 157 mg kg^−1^, respectively. The permanent grassland field had a moderately acidic soil reaction (pH_KCl 5.5) and extractable P_2_O_5_ and K_2_O concentrations of 29 and 99 mg kg^−1^, respectively. Soil organic carbon concentrations were 1.32% in the sown ley and 1.50% in the permanent grassland. The corresponding calculated humus concentrations were 2.28% and 2.58%, respectively. Total nitrogen was not determined.

Extractable phosphorus and potassium were determined and expressed as P_2_O_5_ and K_2_O, respectively, using the Egner–Riehm–Domingo ammonium lactate method. Soil reaction was determined in a KCl suspension according to LST ISO 10390:2005 [45]. Soil organic carbon was determined according to ISO 10694:1995 [46], and humus concentration was calculated by multiplying the soil organic carbon concentration by a conversion factor of 1.724. Because only two composite soil samples were analysed per field, the soil data were treated as descriptive and no inferential statistical comparisons between fields were performed. Neither field was fertilised during the study.

Detailed information on study-area mapping, sampling design, plot codes, grassland assignments, and WGS84 coordinates is provided in Supplementary File S5; the spatial datasets used for study-area mapping are described in [47,48].

The permanent grassland was a fenced 3.62 ha field managed by mowing and low-intensity grazing. It was classified in agricultural declarations as permanent grassland maintained for at least 5 years. Its longer continuity was further supported by the national inventory of habitats of European Community importance conducted in 2011–2015 and by subsequent orthophotographic imagery, which showed no indication that the field had been ploughed or reseeded before or during the study period. The field was located within the Natura 2000 BAST site LTKED0027. Most sampling points were situated within areas mapped during the national habitat inventory as Annex I habitat types 6270* or 6450, while one sampling point was located within the BAST site but outside the mapped Annex I habitat polygons [49]. Pl@ntBERT was trained predominantly on vegetation-plot data from the European Vegetation Archive (EVA) [50,51]. Pl@ntBERT-based EUNIS classification was performed at level 3 using the official model snapshot (Git commit ef1812f) [50] with the EUNIS habitat classification used as the reference framework [52]. Because the 1 × 1 m microplots were smaller than the grassland plots typically represented in the European Vegetation Archive (EVA) training data, this domain mismatch limited the interpretation to descriptive fine-scale floristic affinity and classification consistency. Model details, prediction outputs and limitations, including the absence of confidence scores, are provided in Supplementary File S6.

The sown ley was a fenced 1.85 ha field established in 2017 after wheat and oat cultivation as part of a ley–arable rotation on an organically managed farm. It was sown with a commercial forage grass–legume seed mixture containing *Trifolium pratense* L., *T. repens* L., *Medicago sativa* L., *Festuca pratensis* Huds., *F. arundinacea* Schreb., × *Festulolium* Asch. & Graebn., *Lolium perenne* L., *Poa pratensis* L., *Agrostis gigantea* Roth, *L. multiflorum* Lam., and *Phleum pratense* L. The mixture was applied at a bulk sowing rate of 18 kg ha^−1^, equivalent to 16.9 kg pure live seed ha^−1^, based on 99% seed purity and 95% germination. Records of the cultivars and the proportions of individual species by seed weight were not retained and could not be reconstructed reliably. No mineral or organic fertilisers were applied to either the sown ley or the permanent grassland during the study period. In the autumn of the ley establishment year, the sown ley was mown, and the harvested biomass was removed from the field.

Vegetation composition was recorded before above-ground biomass was harvested from the same 1 m × 1 m quadrats distributed across both grasslands. In total, 29 quadrats were sampled: 16 in the sown ley and 13 in the permanent grassland. Quadrats were positioned using constrained random sampling, with a minimum distance of 10 m maintained from field boundaries and between neighbouring quadrats. Sampling was carried out in mid-June, when the dominant grasses had reached the heading stage. Before biomass harvesting, all vascular plant species present in each quadrat were recorded, and the cover of each species was estimated using the six-grade semi-quantitative Braun–Blanquet cover-abundance scale. Plant nomenclature was standardised according to Euro + Med PlantBase [53]. Plot-level vegetation, functional-trait, EUNIS-classification, biomass-yield, botanical-composition, and herbage-chemistry data are available in the deposited dataset described in the Data Availability Statement.

### 4.2. Animals, Grazing Management and Slaughter

In each study year, eight Aubrac bulls aged 11–12 months were included in two grazing groups, with four animals per group. Thus, 16 bulls were studied across the two years. Until weaning at approximately 8–9 months of age, the calves remained with their dams, suckled freely, and grazed alongside them. Following weaning and during the winter housing period, all bulls were kept together and fed hay and haylage produced from rotational and natural grasslands outside the study fields. From 2 May until the initial weighing and placement in the grazing fields on 18 May, all bulls were kept together on pasture and additionally received hay. No concentrates were provided at any stage from birth to slaughter. Pre-experimental feeding management was the same in 2019 and 2020.

Immediately after the initial weighing, the bulls were placed in their assigned ley or permanent grassland study fields, where they remained throughout the nominal 130-day study period, defined by inclusive counting of the calendar dates from 18 May to 24 September. During the initial 30-day adaptation phase, the bulls grazed continuously in their assigned fields and additionally received hay under the same management conditions in both groups. During the subsequent 100-day monitored grazing phase, from 17 June to 24 September inclusive, supplementary feeding was discontinued, and the bulls relied exclusively on grazing. No hay, haylage, other supplementary feed, or mineral supplements were provided during this phase. Drinking water was available ad libitum throughout the study period.

Bulls were weighed in the morning using the same equipment. Initial liveweight was recorded at the beginning of the 30-day adaptation phase, and final liveweight was recorded at the end of the subsequent 100-day monitored grazing phase. Each bull was weighed once at each time point. Total liveweight change was calculated as final minus initial liveweight, and average daily liveweight change was calculated by dividing total liveweight change by the nominal 130-day study period, defined by inclusive counting of the calendar dates from 18 May to 24 September. Gastrointestinal fill was not measured, and no shrink adjustment was applied; therefore, the calculated values represent average daily liveweight change rather than intake-adjusted tissue gain.

Each bull was considered equivalent to 0.6 livestock units (LU), giving 2.4 LU per treatment group in each grazing season. Based on the effective grazing area, the stocking rate was 0.66 LU ha^−1^ in the permanent grassland and 1.30 LU ha^−1^ in the sown ley. Based on the mean of each animal’s initial and final liveweights, the corresponding mean liveweight-based stocking densities across the two grazing seasons were 561 kg liveweight ha^−1^ in the permanent grassland and 1115 kg liveweight ha^−1^ in the sown ley.

Initial standing herbage DM per bull per nominal grazing day (kg DM bull^−1^ d^−1^) was calculated by multiplying the mid-June herbage DM yield (kg DM ha^−1^) by the effective grazing area (ha) and dividing the resulting total herbage DM by the product of the number of bulls and the nominal 100-day grazing duration. This denominator represented the monitored grazing phase only and differed from the nominal 130-day study period used to calculate average daily liveweight change, which also included the preceding 30-day adaptation phase.

The resulting arithmetic values were 13.5 and 13.1 kg DM bull^−1^ per nominal day of the 100-day monitored grazing phase in the ley and permanent grassland fields, respectively. These values represent neither measured daily herbage allowance nor dry-matter intake. They exclude herbage regrowth, residual biomass, grazing losses, trampling, senescence, diet selection, and temporal changes in herbage availability.

At the end of each grazing season, all bulls were slaughtered at the same approved slaughterhouse following standard commercial procedures, and meat quality was subsequently assessed. Hot carcass weight was recorded shortly after slaughter. The cold carcass weight reported in this study was derived by applying a 2% deduction to the hot carcass weight, while dressing percentage was calculated as hot carcass weight divided by final liveweight × 100. Cold carcass weight, dressing percentage, and EUROP conformation and fat classes were obtained from the slaughterhouse records for all animals. The complete individual-animal records are available in the publicly accessible MIDAS dataset [54]. The privately owned animals were managed under routine commercial farming conditions. No additional invasive procedures or interventions beyond standard farm management were undertaken for research purposes.

### 4.3. Functional Diversity and Ecological Indicator Analysis

Functional diversity was quantified at the plot level using a multidimensional trait-based framework. Each 1 m^2^ vegetation plot was treated as a community sampling unit, resulting in 16 ley and 13 permanent grassland plots. This approach retained within-type variation among plots rather than reducing each grassland type to a single pooled community.

Functional traits were selected to represent persistence, regeneration and dispersal strategies. Life-form categories were obtained from the FloraVeg.eu life-form database and harmonised into combined categorical life-form states, allowing species with more than one life-form strategy to retain combined categories following Dřevojan et al. [55]. Seed mass, predominant dispersal mode and dispersal-distance class were obtained from the European flora seed-dispersal dataset of Lososová et al. [56]. Seed mass was log10-transformed as log10 (seed mass + 1), dispersal-distance class was treated as an ordered trait, and dispersal mode and life form were treated as categorical traits.

Four complementary metrics were calculated: functional richness (FRic), Rao’s quadratic entropy (RaoQ), functional dispersion (FDis) and functional evenness (FEve). FRic was calculated as the convex-hull volume of the occupied functional trait space without abundance weighting, whereas RaoQ, FDis and FEve were weighted by relative plant cover.

Taxonomic harmonisation between vegetation records and trait databases was applied where necessary, with aggregate-level trait values used when species-level matching was not possible.

Trait coverage was evaluated before functional diversity was calculated. Complete trait data were available for 88 of the 90 vegetation taxa, representing 97.78% of the taxon pool and 98.68% of cumulative vegetation cover. Coverage was complete in the ley and reached 97.84% of cumulative vegetation cover in the permanent grassland. Only *Equisetum arvense* and *E. palustre* were excluded, together representing 1.32% of total cumulative cover.

Plot-level ecological indicator profiles were calculated using nine indicators: light, temperature, moisture, soil reaction and nutrients from the European Ellenberg-type indicator dataset [57], and disturbance severity, disturbance frequency, mowing frequency and grazing pressure from the European disturbance-indicator dataset [58]. These indicators were used to characterise the ecological affinities of co-occurring species along resource, environmental and disturbance gradients. The indicators were interpreted as community-level ecological profiles, not as direct measurements of soil chemistry, microclimate or experimentally quantified species response curves. Community-weighted means were calculated for each quadrat using species cover values.

### 4.4. Biomass Yield and Botanical Group Composition

Vegetation and biomass sampling was conducted once, in mid-June, during the grazing period. The sampling quadrats were not protected from grazing by exclusion cages. Consequently, the botanical-composition, biomass-yield, and chemical-composition data characterise the standing herbage after approximately 30 days of grazing rather than ungrazed baseline conditions or the forage selected and consumed by the animals throughout the grazing period. Subsequent herbage regrowth, seasonal changes in forage availability and composition, residual biomass, grazing losses, and individual-animal intake were not measured.

Following the vegetation survey, above-ground biomass was harvested from each 1 m^2^ quadrat at a stubble height of approximately 5 cm. Fresh biomass was weighed immediately and expressed as g m^−2^. The harvested biomass was separated into grasses, legumes, forbs, and sedges, and each botanical fraction was weighed. The proportion of each botanical group was calculated separately within each quadrat as the mass of the corresponding fraction divided by the total quadrat biomass. The fractions were then recombined and thoroughly homogenised within each quadrat. A representative 500 g subsample was collected from the homogenised biomass for DM determination, and separate representative subsamples were retained for forage-composition and phenolic and antioxidant analyses.

The subsample used for DM determination was pre-dried at 105 °C for 15 min and subsequently dried at 65 ± 5 °C for 24 h. DMY for each quadrat was calculated by multiplying FMY by the corresponding DM proportion. FMY and DMY were expressed as t ha^−1^.

### 4.5. Chemical Analyses

#### 4.5.1. Plant Biomass Chemical Analyses

Fresh plant material was cut into 3–5 cm pieces, pre-dried at 105 °C for 15 min, and subsequently dried in an oven at 65 ± 5 °C for 24 h. The dried material was ground in a cyclone mill equipped with a 1 mm sieve and stored in sealed containers until analysis.

Herbage chemical composition was assessed by estimating crude protein (CP), crude fat (CF), acid detergent fibre (ADF), and neutral detergent fibre (NDF) using a near-infrared spectrometer (NIRS 6500, FOSS NIRSystems, Laurel/Silver Spring, MD, USA) and forage-calibration equations developed for grass and legume forage materials. The calibration sets included 862, 530, 322, and 284 samples for CP, CF, ADF, and NDF, respectively, with cross-validated R^2^ values of 0.97, 0.81, 0.92, and 0.92 and corresponding standard errors of cross-validation of 0.58, 0.29, 1.02, and 1.71 percentage points of DM, respectively [59]. Reference values were obtained using the Kjeldahl method for CP, Soxhlet extraction for CF, and the Van Soest detergent-fibre method for ADF and NDF. Sample-level global and neighbourhood H statistics and independent reference-method measurements were unavailable for the present samples. These constituents are therefore reported as NIRS estimates. The transferability of the ADF and NDF calibrations to the forb-rich permanent grassland biomass could not be independently verified. Results were expressed as percentages of DM.

Separate fresh-herbage subsamples intended for phenolic and antioxidant analyses were frozen and subsequently freeze-dried. The freeze-dried material was ground and extracted with 70% aqueous ethanol at a sample-to-solvent ratio of 1:25 (*w*/*v*) for 24 h in the dark at room temperature, following the procedure described by Puzerytė et al. [60]. The extracts were subsequently centrifuged and filtered before analysis.

TPC was determined using the Folin–Ciocalteu method, with gallic acid as the calibration standard. Absorbance was measured at 750 nm using a GENESYS 10S UV–Vis spectrophotometer (Thermo Scientific, Waltham, MA, USA). Measurements were performed in triplicate, and TPC was expressed as milligrams of gallic acid equivalents per gram of dry weight (mg GAE g^−1^ DW).

DPPH radical-scavenging activity was determined according to the method of Brand-Williams et al. [61], with modifications described previously [62]. A 20 µL aliquot of each sample extract was mixed with 2 mL of DPPH solution prepared in 99% ethanol. After incubation for 30 min in the dark at room temperature, absorbance was measured at 515 nm using a Cintra 202 spectrophotometer (GBC Scientific Equipment, Knox, Australia). DPPH radical-scavenging activity was calculated using a Trolox calibration curve and expressed as micromoles of Trolox equivalents per gram of dry weight (µmol TE g^−1^ DW).

#### 4.5.2. Meat Chemical Composition and Physical Properties

After the carcasses had been stored for 48 h at 4 °C in the slaughterhouse chilling room, a 600 g sample of the *longissimus* muscle was collected immediately posterior to the 13th rib from each Aubrac beef carcass for the determination of meat physicochemical properties, proximate composition, and fatty acid profile. The same anatomical sampling position was used in both years. The collected muscle samples were transported to the laboratory under chilled conditions and prepared according to the requirements of each analytical procedure.

Meat pH was measured 48 h after slaughter using a Hanna HI-2002-02 pH meter (Hanna Instruments Inc., Woonsocket, RI, USA) equipped with a stainless steel electrode according to the LST ISO 2917:2002 [63], which is identical to ISO 2917:1999 [64]. The pH meter was calibrated using standard buffer solutions at pH 4.0 and 7.0. The mean value of active acidity (pH) was calculated from three replicate measurements.

Meat colour measurement generally followed the American Meat Science Association guidelines [65]. Measurements were taken on the exposed cut surface of the lean muscle after 30 min of blooming using a CR-400 colourimeter (Konica Minolta, Inc., Osaka, Japan) in the CIE L*a*b* colour space with illuminant D65, a 2° standard observer, and an 8 mm measurement aperture. The instrument was calibrated against the same standard white calibration tile before each measurement session. The same instrument, measurement settings, calibration procedure, sample preparation, and blooming time were used in 2019 and 2020.

Dry matter content was determined according to the LST ISO 1442:2000 [66] reference method. Samples were mixed with pre-dried sand and dried in a laboratory oven at 103 ± 2 °C until constant mass. Dry matter content was calculated from the mass remaining after drying and expressed as a percentage of the initial sample mass. All weighings before and after drying were performed using the analytical balance incorporated in the MA 50/1.R system (RADWAG, Radom, Poland) under ambient laboratory conditions (20 ± 1 °C).

Drip loss was determined using the standard bag method described by Honikel [67]. A standardised muscle sample weighing 40–50 g and measuring approximately 30 × 60 × 25 mm was suspended in an airtight container at 4 °C for 48 h. Drip loss was expressed as the percentage decrease in sample weight relative to its initial weight.

Cooking loss was determined according to the Schilling method [68]. Approximately 30 g of meat was cut perpendicular to the muscle fibre direction, weighed, vacuum-sealed in a heat-resistant bag, and cooked in a 72 °C water bath until the core temperature reached 70 °C. Samples were then cooled to room temperature, blotted with filter paper, reweighed, and the cooking loss was expressed as a percentage of the initial weight.

Water-holding capacity was determined using the Hamm press method [69]. IMF content was determined by Soxhlet extraction (AOAC 960.39), protein content by the Kjeldahl method (AOAC 928.08), and ash content by incineration at 550 °C to a constant weight (AOAC 920.153) [70].

The fatty acid composition of IMF was determined by gas chromatography following conversion to fatty acid methyl esters (FAME). The samples were prepared according to ISO 12966-2:2017 [71]. Chromatographic analysis of FAME was performed using a Shimadzu GC-2010 Plus system (Shimadzu Corporation, Kyoto, Japan) equipped with a flame ionisation detector and a BPX-70 capillary column (120 m × 0.25 mm internal diameter, 0.25 µm film thickness), following the methodology specified in LST EN ISO 12966-4 [72]. The column oven temperature was maintained at 60 °C for 2 min, increased at 20 °C min^−1^ to 230 °C, and maintained at 230 °C for 45 min. The injector and detector temperatures were 250 °C and 270 °C, respectively. Helium was used as the carrier gas.

Fatty acid methyl esters were identified by comparing sample peak retention times with those of the Supelco 37 Component FAME Mix (Catalog No. 47885-U; Supelco, Bellefonte, PA, USA). No additional reference standards for individual trans-C18:1 positional isomers were used. The reference mixture contained methyl elaidate (C18:1 trans-9) and methyl linolelaidate (C18:2 trans-9,trans-12) but did not contain C18:1 trans-10 or vaccenic acid (C18:1 trans-11). The same chromatographic, peak-identification, and integration procedures were applied to all samples analysed in 2019 and 2020.

Fatty acid values were expressed as percentages of total identified fatty acids. Before statistical analysis, individual fatty acids were assigned to predefined biochemical classes, and the classification was checked for consistency between the two sampling years. All fatty acids identified as n-3 or n-6 were assigned to the PUFA class. Rumenic acid (CLA cis-9,trans-11) was not quantified or included as a separately identified component in the reported PUFA, n-6 PUFA, or trans FA sums. The PUFA and n-6 PUFA totals were calculated from the individual components assigned as cis-PUFA in the deposited animal-level dataset, whereas the trans FA total comprised the unresolved trans-C18:1 component and the component assigned to C18:2 trans-9,trans-12.

Because no additional standards for individual trans-C18:1 positional isomers were used, the component originally assigned to elaidic acid could not be confirmed as representing C18:1 trans-9 alone. The possible contribution of other co-eluting trans-C18:1 positional isomers, including vaccenic acid (C18:1 trans-11), could not be excluded. This component was therefore conservatively reported as unresolved trans-C18:1 fatty acids. Linolelaidic acid (C18:2 trans-9,trans-12) was identified by comparison with the corresponding component of the reference mixture.

The original chromatograms, retention-time tables, and contemporaneous peak-identification records were unavailable for retrospective verification. Consequently, individual trans-C18:1 positional isomers could not be re-examined or quantified separately, and vaccenic acid was not reported as an individual fatty acid. Similarly, the separate identification and quantification of rumenic acid could not be verified. It therefore remains uncertain whether rumenic acid was absent or whether an unresolved contribution may have been incorporated into another chromatographic component. The absence of a separately quantified CLA component should not be interpreted as evidence that rumenic acid was absent from the samples.

Saturated fatty acids (SFA), cis-monounsaturated fatty acids (cis-MUFA), polyunsaturated fatty acids (PUFA), trans fatty acids (trans FA), n-3 PUFA, and n-6 PUFA were calculated for each animal by summing the individual fatty acids assigned to the corresponding predefined class. Unresolved trans-C18:1 fatty acids and the identified trans-C18:2 component were assigned exclusively to the trans FA class and were not included in the cis-MUFA or PUFA sums. The PUFA/SFA and n-6/n-3 ratios were calculated at the individual-animal level. Individual fatty acid values by grassland group and year are provided in Supplementary File S3.

Lipid quality indices were calculated for each animal before statistical analysis and group-level summarisation. The atherogenicity index (AI) and thrombogenicity index (TI) were calculated according to Ulbricht and Southgate [40], and the hypocholesterolaemic/hypercholesterolaemic fatty acid ratio (h/H) was calculated according to Santos-Silva et al. [73]. Trans fatty acids were excluded from the sums of unsaturated fatty acids used in these calculations.

### 4.6. Statistical Analysis

Vegetation quadrats and biomass samples were sampling units within each field, whereas individual bulls were animal-level observations within each field × year combination. Because one ley and one permanent grassland field were studied, analyses quantify field-specific contrasts and do not provide independent field-level replication. Different bulls were studied in 2019 and 2020.

Taxonomic diversity was analysed using incidence-based Hill numbers of orders q = 0, q = 1, and q = 2, representing species richness, Shannon effective diversity, and Simpson effective diversity, respectively. Each 1 m^2^ vegetation plot was treated as a sampling unit, and species presence or absence in each plot was used to construct the incidence data. Because observed sample coverage differed between the two fields, the primary comparison was standardised to a common sample coverage of 91.50%, corresponding to the lower observed coverage. Coverage-based diversity estimates and 95% bootstrap confidence intervals were calculated using the iNEXT package with 9999 bootstrap replications [74,75]. Observed and asymptotic diversity estimates were evaluated separately and are reported in Supplementary File S1.

Functional diversity was calculated per quadrat using Gower trait distances and the dbFD function in the FD package [76,77]. RaoQ, FDis, and FEve were weighted by relative plant cover, whereas FRic was unweighted and treated as exploratory because of poor reduced-space representation. Between-field differences were tested using two-sided permutation tests of mean differences with 9999 permutations. Plot-level community-weighted ecological-indicator profiles were centred and scaled, visualised by PCA, and compared using one-way PERMANOVA based on Euclidean distances; PERMDISP assessed multivariate dispersion. The indicator analysis was repeated using high-coverage indicators as a robustness check [78,79].

The eight prespecified biomass-yield and chemistry outcomes were FMY, DMY, CP, CF, ADF, NDF, TPC, and DPPH. Outcomes are reported as the mean ± SD. Approximate normality was assessed within each field using the Shapiro–Wilk test. For outcomes for which approximate normality was adequately supported in both fields, between-field comparisons used Welch’s *t*-test, thereby avoiding the need to assume equal population variances. When approximate normality was not adequately supported in at least one field, the Wilcoxon rank-sum test was used. Variance heterogeneity was assessed using the median-centred Levene test, with the Fligner–Killeen test as an additional robust diagnostic, but these diagnostics were not used to select between pooled and unequal-variance *t*-tests. Benjamini–Hochberg FDR correction was applied across the eight primary *p*-values, and significance was assessed at q < 0.05 [80]. Two-sided permutation tests of mean differences, yield coefficients of variation, and Fligner–Killeen dispersion tests were treated as sensitivity or complementary analyses and were not included in this FDR family.

Botanical-group percentages were calculated within each quadrat and averaged descriptively across quadrats. The four proportions were Hellinger-transformed and evaluated jointly using one-way PERMANOVA based on Euclidean distances, with PERMDISP used to assess multivariate dispersion [78,79]. The integrated profile of TPC, DPPH, CP, CF, ADF, NDF, FMY, and DMY was assessed using PCA after centring and scaling, followed by PERMANOVA and PERMDISP with 9999 permutations [78,79].

Animal growth is reported as the mean ± SD. Cold carcass weight and dressing percentage were summarised descriptively for each studied field × year combination as the means ± SD. EUROP conformation and fat classes were summarised as counts. No inferential statistical tests were performed for the carcass characteristics because each studied field × year combination included only four animals. Initial liveweight was compared descriptively between groups within each year using Welch’s *t*-tests and exact two-sided permutation tests. Final liveweight was analysed by ANCOVA with initial liveweight as a covariate and studied field and year as fixed terms. A studied field × year interaction was examined separately as an exploratory model. Statistical significance of partial F statistics was evaluated using Freedman–Lane permutation tests with 9999 permutations [81].

Physicochemical and proximate-composition outcomes were analysed using animal-level two-way linear models containing group, year, and group × year. Significance was evaluated using Freedman–Lane permutation tests with 9999 permutations [81]. The physicochemical-property family comprised seven outcomes (pH, L*, a*, b*, drip loss, water-holding capacity, and cooking loss), whereas the proximate-composition family comprised four outcomes (dry matter, intramuscular fat, ash, and protein). Benjamini–Hochberg correction was applied separately within each outcome family and separately for the group, year, and group × year model terms [80].

For fatty acid analysis, SFA, cis-MUFA, PUFA, trans FA, n-3 PUFA, and n-6 PUFA were analysed as logit-transformed proportions and back-transformed to percentages. These outcomes were modelled marginally; because they are components of a fixed total, their contrasts are compositionally constrained and were interpreted as related shifts in relative composition rather than independent changes in absolute concentrations. The n-6/n-3 ratio was analysed after natural-log transformation and reported as geometric marginal means with the permanent grassland-to-ley ratio as the contrast. PUFA/SFA, AI, TI, and h/H were analysed on their original scales. Models included group, year, and group × year; marginal group estimates gave equal weight to both years.

The fatty acid and lipid-quality family comprised 11 prespecified outcomes. Benjamini–Hochberg correction was applied separately across the 11 *p*-values for each of the group, year, and group × year terms [80]. Because the class proportions are compositionally constrained and several ratios and indices are algebraically derived from the same fatty acid profile, q-values were interpreted together with effect estimates and 95% confidence intervals. Models were additionally refitted with IMF as a covariate, and the group term was evaluated using Freedman–Lane permutation tests as a sensitivity analysis [81].

Model assessment combined graphical residual diagnostics, residual Shapiro–Wilk tests, Cook’s distance, and median-centred Levene tests. All tests were two-sided. Analyses were conducted in R version 4.5.2 [82], using iNEXT [75], FD [77], vegan [79], tidyverse packages [83], and ggplot2 [84]. Full diagnostics and supporting outputs are provided in the Supplementary Files and deposited datasets.

## Figures and Tables

**Table 1:** Plot-level functional diversity metrics based on persistence, regeneration, and dispersal traits in the permanent grassland and sown ley fields.

Metric	Ley (*n* = 16) Mean ± SD	Permanent Grassland (*n* = 13) Mean ± SD	Mean Difference	Permutation *p*-Value
FDis	0.053 ± 0.052	0.111 ± 0.039	0.0583	0.0026
RaoQ	0.008 ± 0.009	0.023 ± 0.008	0.0150	0.0002
FEve	0.533 ± 0.149	0.590 ± 0.070	0.0574	0.2151

Note: RaoQ, Rao’s quadratic entropy; FDis, functional dispersion; FEve, functional evenness. The metrics were calculated using life form, log-transformed seed mass, predominant dispersal mode, and dispersal-distance class and therefore represent persistence, regeneration, and dispersal-strategy dimensions. Mean differences were calculated as permanent grassland minus ley; positive values indicate higher values in the permanent grassland. *p*-values were obtained from two-sided permutation tests of differences between group means based on 9999 permutations. FRic was excluded from the primary inferential comparison because of the low quality of the reduced-space representation (qual.FRic = 0.284) and is reported only as an exploratory metric, with the corresponding reduced-space diagnostics provided in Supplementary File S2.

**Table 2:** Biomass yield and chemical characteristics of the studied ley and permanent grassland fields.

Outcome	Unit	Ley, Mean ± SD (*n* = 16)	Permanent Grassland, Mean ± SD (*n* = 13)	Test	Statistic	Unadjusted *p*	FDR q
FMY	t ha^−1^	12.91 ± 7.01	6.68 ± 4.10	Wilcoxon rank-sum	W = 150.0	0.0460	0.0613
DMY	t ha^−1^	2.92 ± 1.56	1.45 ± 0.86	Wilcoxon rank-sum	W = 163.0	0.0103	0.0165
CP	% DM	14.85 ± 1.12	15.84 ± 3.06	Welch *t*-test	*t* = −1.105	0.2871	0.3281
CF	% DM	1.57 ± 0.27	1.55 ± 0.55	Welch *t*-test	*t* = 0.174	0.8637	0.8637
ADF	% DM	29.40 ± 3.97	34.91 ± 5.46	Wilcoxon rank-sum	W = 35.5	0.00285	0.00569
NDF	% DM	47.60 ± 6.08	57.94 ± 7.94	Wilcoxon rank-sum	W = 33.5	0.00214	0.00569
TPC	mg GAE g^−1^ DW	13.58 ± 3.65	22.33 ± 8.13	Welch *t*-test	*t* = −3.598	0.00243	0.00569
DPPH	µmol TE g^−1^ DW	57.19 ± 11.01	141.70 ± 63.20	Welch *t*-test	*t* = −4.763	0.000404	0.00323

Note: Values are presented as the mean ± SD. FMY, fresh-matter yield; DMY, dry-matter yield; CP, crude protein; CF, crude fat; ADF, acid detergent fibre; NDF, neutral detergent fibre; TPC, total phenolic content; DPPH, DPPH radical-scavenging activity; GAE, gallic acid equivalents; TE, Trolox equivalents; DM, dry matter; DW, dry weight. Tests were selected according to the decision rule described in Section 4.6: Wilcoxon rank-sum tests were applied when approximate normality was insufficiently supported in at least one group, whereas Welch’s *t*-tests were used for parametric two-group comparisons when approximate normality was adequately supported in both groups. Reported *p*-values are unadjusted, whereas q-values were obtained using the Benjamini–Hochberg false-discovery-rate correction across the eight prespecified yield and biomass-chemistry outcomes.

**Table 3:** Botanical-group composition of the studied ley and permanent grassland fields.

Botanical Group	Ley, %, Mean ± SD (*n* = 16)	Permanent Grassland, %, Mean ± SD (*n* = 13)
Legumes	30.86 ± 14.90	7.11 ± 4.79
Grasses	68.56 ± 15.69	40.38 ± 16.21
Forbs	0.59 ± 1.63	46.73 ± 19.38
Sedges	0.00 ± 0.00	5.79 ± 12.59

Note. Values are percentages of within-quadrat botanical biomass and are presented as the arithmetic mean ± SD across quadrats. The four proportions were analysed jointly using PERMANOVA because they are interdependent and sum to 100%; therefore, separate univariate tests and FDR-adjusted q-values were not calculated. Full analytical details are provided in Supplementary File S2.

**Table 4:** Growth and carcass characteristics of bulls in the ley and permanent grassland grazing groups in 2019 and 2020.

Year	Grassland	*n*	Initial Liveweight (kg)	Final Liveweight (kg)	Total Liveweight Change (kg)	Average Daily Liveweight Change (kg d^−1^)	Cold Carcass Weight (kg)	Dressing Percentage (%)
2019	Ley	4	435.3 ± 84.8	600.3 ± 61.5	165.0 ± 49.4	1.27 ± 0.38	342.1 ± 34.4	58.3 ± 1.3
2019	Permanent grassland	4	440.5 ± 72.9	579.8 ± 28.1	139.3 ± 65.0	1.07 ± 0.50	319.6 ± 19.6	56.3 ± 1.5
2020	Ley	4	446.3 ± 32.7	581.8 ± 5.4	135.5 ± 36.3	1.04 ± 0.28	322.1 ± 12.5	56.3 ± 1.5
2020	Permanent grassland	4	431.0 ± 47.1	578.5 ± 13.5	147.5 ± 35.2	1.13 ± 0.27	307.5 ± 2.4	54.0 ± 1.2

Note: Values are means ± SD. Cold carcass weight, dressing percentage, and EUROP conformation and fat classes were obtained from slaughterhouse records. Cold carcass weight was derived by applying a 2% deduction to hot carcass weight. Dressing percentage was calculated as hot carcass weight divided by final liveweight × 100. EUROP classifications were summarised descriptively; overall, 11 of 16 carcasses were assigned to conformation class U and 14 of 16 to fat class 2. Average daily liveweight change was calculated by dividing total liveweight change by the nominal 130-day study period, defined by inclusive counting of the calendar dates from 18 May to 24 September. This period included 30 days of hay supplementation and therefore does not represent a grazing-only phase. The quantity of supplementary hay offered during the 30-day adaptation phase was not recorded. Each animal was weighed once at the beginning and once at the end of this period. Gastrointestinal fill was not measured, and no shrink adjustment was applied. Different animals were studied in 2019 and 2020, with four animals included in each grassland system in each year.

**Table 5:** Physicochemical properties and proximate composition of the *longissimus* muscle from bulls in the ley and permanent grassland groups in 2019 and 2020.

Parameter	2019		2020		FDR-Adjusted *p*-Value		
	Ley	Permanent Grassland	Ley	Permanent Grassland	qG	qY	qG × Y
Dry matter, %	27.70 ± 1.38	27.28 ± 2.12	28.07 ± 1.60	28.10 ± 1.83	0.882	0.651	0.817
pH	5.63 ± 0.03	5.63 ± 0.05	5.62 ± 0.08	5.70 ± 0.12	0.980	0.766	0.778
Lightness (L*)	37.11 ± 2.66	38.41 ± 1.57	37.50 ± 2.91	38.14 ± 1.64	0.980	0.960	0.778
Redness (a*)	17.56 ± 0.68	19.14 ± 1.13	26.13 ± 5.66	24.13 ± 4.61	0.980	0.035	0.778
Yellowness (b*)	8.54 ± 2.29	9.18 ± 1.99	14.64 ± 5.44	12.70 ± 4.72	0.980	0.091	0.778
Drip loss, %	2.34 ± 1.82	2.49 ± 1.26	1.59 ± 0.88	1.29 ± 0.88	0.980	0.260	0.778
Water-holding capacity, %	57.45 ± 1.04	56.45 ± 1.23	56.63 ± 3.42	56.95 ± 4.49	0.980	0.960	0.778
Cooking loss, %	26.08 ± 2.15	28.75 ± 1.78	26.40 ± 4.38	22.99 ± 3.21	0.980	0.260	0.462
Intramuscular fat, %	1.94 ± 0.57	1.65 ± 0.38	3.73 ± 0.49	3.76 ± 0.67	0.882	0.002	0.817
Ash, %	1.07 ± 0.05	1.04 ± 0.16	1.04 ± 0.17	1.05 ± 0.07	0.882	0.884	0.817
Protein, %	24.70 ± 0.93	24.59 ± 1.98	24.04 ± 0.80	23.29 ± 1.30	0.882	0.314	0.817

Note: Values are means ± SD. For each year, *n* = 4 per grazing group. qG, qY and qG × Y are Benjamini–Hochberg FDR-adjusted permutation *p*-values for the group, year and group × year terms, respectively. FDR correction was applied separately within the seven-outcome physicochemical-property family and the four-outcome proximate-composition family, and separately for each model term; q < 0.05 was considered statistically significant.

**Table 6:** Model-adjusted intramuscular fatty acid proportions, fatty acid ratios and lipid quality indices in young bulls from the ley and permanent grassland groups.

Outcome	Ley	Permanent Grassland	Group Contrast	q
SFA, %	50.19 (49.63 to 50.75)	44.46 (43.90 to 45.03)	−5.73 (−6.53 to −4.90)	<0.001
cis-MUFA, %	35.01 (33.73 to 36.29)	38.41 (37.07 to 39.76)	3.40 (1.54 to 5.34)	0.006
PUFA, %	9.84 (8.75 to 11.06)	12.84 (11.46 to 14.38)	3.00 (1.12 to 4.95)	0.006
trans FA, %	4.87 (4.29 to 5.53)	4.06 (3.58 to 4.63)	−0.80 (−1.63 to 0.03)	0.104
n-3 PUFA, %	2.69 (2.15 to 3.41)	3.98 (3.22 to 4.96)	1.29 (0.25 to 2.43)	0.007
n-6 PUFA, %	7.07 (6.21 to 8.04)	8.81 (7.77 to 10.01)	1.75 (0.30 to 3.26)	0.027
PUFA/SFA	0.196 (0.157 to 0.235)	0.293 (0.254 to 0.332)	0.097 (0.042 to 0.153)	0.005
n-6/n-3	3.006 (2.332 to 3.876)	2.272 (1.762 to 2.929)	0.756 (0.528 to 1.082)	0.115
AI	0.906 (0.854 to 0.958)	0.700 (0.648 to 0.752)	−0.206 (−0.280 to −0.132)	<0.001
TI	1.535 (1.473 to 1.598)	1.096 (1.034 to 1.159)	−0.439 (−0.528 to −0.350)	<0.001
h/H	1.258 (1.201 to 1.314)	1.591 (1.534 to 1.647)	0.333 (0.253 to 0.413)	<0.001

Note: Values are model-adjusted marginal estimates with 95% confidence intervals, obtained by equally weighting the estimates for 2019 and 2020. Percentage-outcome contrasts are expressed as Permanent grassland minus Ley differences in percentage points; contrasts for PUFA/SFA, AI, TI, and h/H are expressed as differences on the original scale. For n-6/n-3, estimates are geometric marginal means and the contrast is the Permanent/Ley ratio. The trans FA class comprised unresolved trans-C18:1 fatty acids and linolelaidic acid (C18:2 trans-9,trans-12); individual trans-C18:1 positional isomers were not quantified separately, and this outcome was treated as exploratory. Reported q-values are Benjamini–Hochberg FDR-adjusted *p*-values for the group effect across 11 prespecified outcomes. Estimates represent animal-level contrasts conditional on the two studied fields. SFA, saturated fatty acids; cis-MUFA, cis-monounsaturated fatty acids; PUFA, polyunsaturated fatty acids; trans FA, trans fatty acids; AI, atherogenicity index; TI, thrombogenicity index; h/H, hypocholesterolaemic-to-hypercholesterolaemic fatty acid ratio.

## Data Availability

The data presented in this study are openly available in the National Open Access Research Data Archive (MIDAS) at https://doi.org/10.18279/MIDAS.560807 (accessed on 6 September 2026) [54] and https://doi.org/10.18279/MIDAS.560532 (accessed on 15 August 2026) [85]. Individual Animal_ID identifiers were used to link the liveweight, carcass, meat-quality, proximate-composition, and fatty acid records. The consistency of these identifiers and the corresponding grazing-group assignments was cross-checked across the deposited animal-level datasets.

## Supplementary Materials

The following supporting information can be downloaded at: https://www.mdpi.com/article/10.3390/plants15193026/s1, Supplementary File S1: Taxonomic Diversity Analysis, including Table S1 and Figures S1 and S2; Supplementary File S2: Analyses of Biomass Yield, Chemistry, Botanical Composition, and Functional Diversity, including Tables S2–S7; Supplementary File S3: Intramuscular fatty acid proportions, including Table S8; Supplementary File S4: Intramuscular Fat Sensitivity Analysis, including Table S9; Supplementary File S5: Study-Area Mapping, Sampling Design, and Plot Coordinates, including Figure S3 and Table S10; Supplementary File S6: Pl@ntBERT-Based EUNIS Classification of Grassland Microplots, including Figure S4 and Table S11.

## Abbreviations

The following abbreviations are used in this manuscript:

ADF	Acid detergent fibre
AI	Atherogenicity index
ANCOVA	Analysis of covariance
BAST	Habitat Protection Area (Lithuanian designation)
CF	Crude fat
CI	Confidence interval
cis-MUFA	cis-Monounsaturated fatty acids
CP	Crude protein
DM	Dry matter
DMY	Dry matter yield
DPPH	2,2-diphenyl-1-picrylhydrazyl
DW	Dry weight
EUNIS	European Nature Information System
EVA	European Vegetation Archive
FAME	Fatty acid methyl esters
FDis	Functional dispersion
FDR	False discovery rate
FEve	Functional evenness
FMY	Fresh matter yield
FRic	Functional richness
GAE	Gallic acid equivalents
h/H	Hypocholesterolaemic/hypercholesterolaemic fatty acid ratio
IMF	Intramuscular fat
LU	Livestock unit
NDF	Neutral detergent fibre
NIRS	Near-infrared spectroscopy
PC1	First principal component
PC2	Second principal component
PCA	Principal component analysis
PERMANOVA	Permutational multivariate analysis of variance
PERMDISP	Permutational analysis of multivariate dispersion
PUFA	Polyunsaturated fatty acids
RaoQ	Rao’s quadratic entropy
SD	Standard deviation
SFA	Saturated fatty acids
TE	Trolox equivalents
TI	Thrombogenicity index
TPC	Total phenolic content
trans FA	Trans fatty acids
WGS84	World Geodetic System 1984
