# Supplementary material for: Comparing Vegetation Characteristics and Production Outcomes of Semi-Natural Permanent Grassland and Sown Ley: A Case Study in a Hemi-Boreal Region
Source: Plants (Basel). 2026 Oct 2;15(19):3026. doi: 10.3390/plants15193026 (PMC13667139; doi:10.3390/plants15193026)
Supplement: Supplementary file 1 [file plants-15-03026-s001.zip › Supplementary_File_S1_Taxonomic Diversity Analysis_Table_S1 Figures_S1 S2.pdf]

Supplementary taxonomic-diversity analysis

Taxonomic diversity was evaluated using incidence-based Hill numbers calculated from species occurrences across 1 m^2 vegetation plots. The primary comparison in the manuscript was standardised to a common sample coverage of 91.5%. This supplementary section presents the observed and asymptotic Hill-number estimates, sample-size-based rarefaction and extrapolation curves, and sample-completeness curves. These outputs distinguish the observed reference-sample results from estimated asymptotic diversity and document the difference in sampling completeness between the two fields.

Table S1. Observed and asymptotic incidence-based Hill diversity in the ley and permanent grassland.

Field	Diversity measure	Observed	Asymptotic estimate	95% CI
Ley	Species richness,	12.00	12.23	12.00–16.30
Ley	Shannon effective diversity,	9.23	9.51	8.53–10.50
Ley	Simpson effective diversity,	8.12	8.26	7.48–9.04
Permanent grassland	Species richness,	84.00	119.29	94.45–144.12
Permanent grassland	Shannon effective diversity,	61.76	72.77	65.68–79.86
Permanent grassland	Simpson effective diversity,	51.08	56.04	51.07–61.00

Note: Observed values describe the reference samples of 16 ley plots and 13 permanent-grassland plots. Asymptotic estimates represent estimated diversity as sample size approaches completeness and were not used as the primary between-field comparison. The primary comparison was standardised to a common sample coverage of 91.50% and is reported in the main text and Figure 1. Confidence intervals were calculated using 9,999 bootstrap replications.

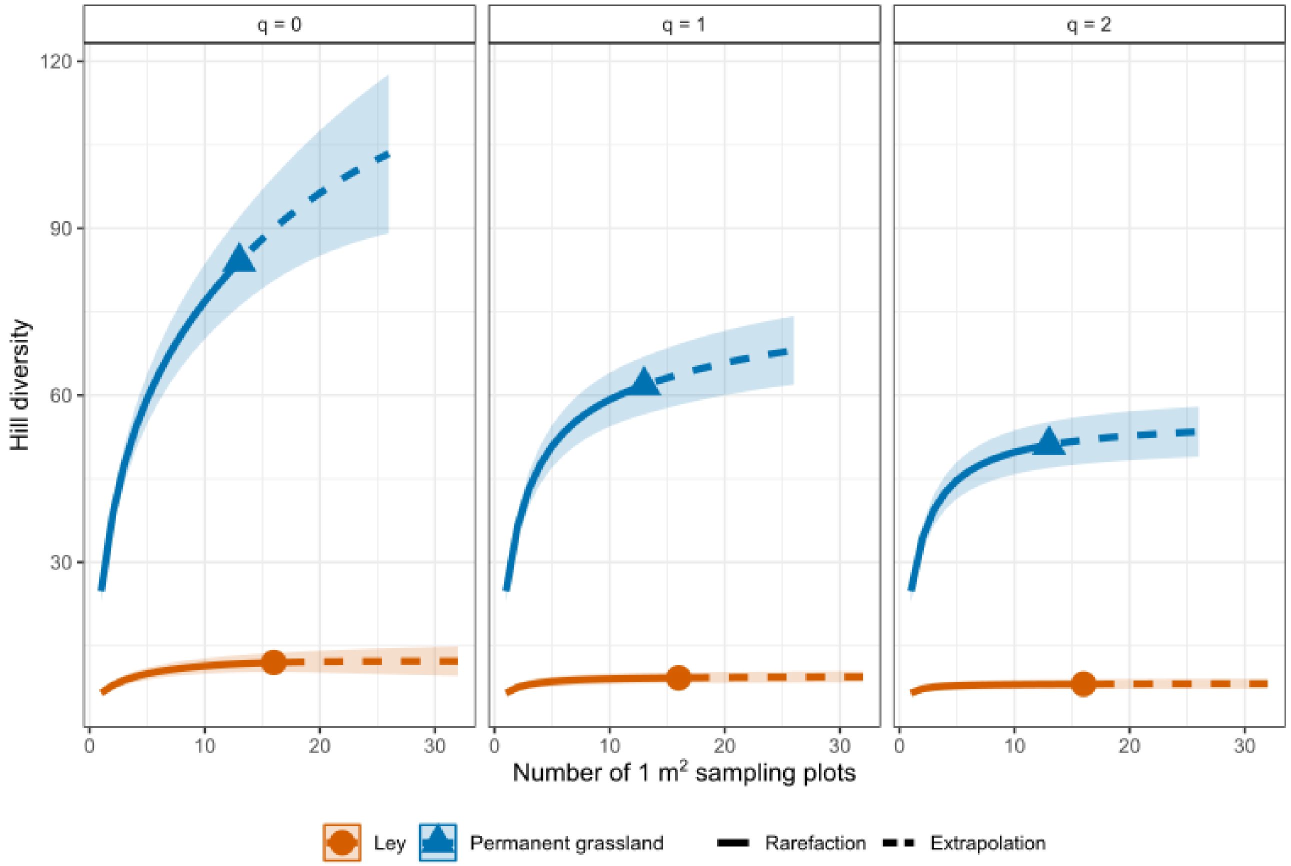

Figure S1. Sample-size-based rarefaction and extrapolation curves for incidence-based Hill diversity in the ley and permanent grassland. Hill numbers of orders q = 0, q = 1, and q = 2 represent species richness, Shannon effective diversity, and Simpson effective diversity, respectively. Sampling units were 1 m^2 vegetation plots, and shaded areas indicate 95% bootstrap confidence intervals calculated using 9,999 bootstrap replications. The curves illustrate how estimated diversity varies with the number of sampling plots; the primary coverage-standardised comparison is presented in the main text.

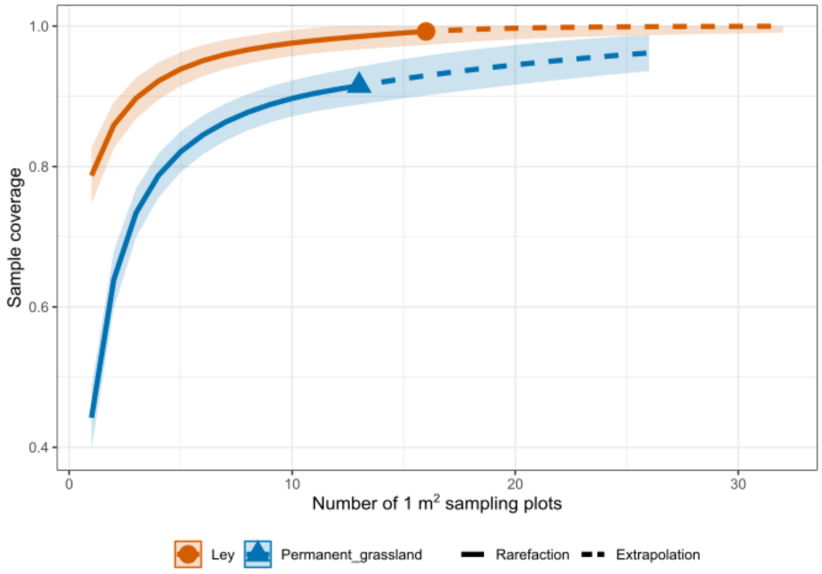

Figure S2. Sample-completeness curves for the ley and permanent grassland based on species incidences across 1 m^2 vegetation plots. Sample coverage represents the proportion of the total incidence probability accounted for by the detected taxa. Observed coverage was 99.24% for the ley and 91.53% for the permanent grassland.

The lower coverage and continuing increase of the richness curve for the permanent grassland indicate that additional sampling would likely have detected further infrequent taxa.
