# Supplementary material for: Comparing Vegetation Characteristics and Production Outcomes of Semi-Natural Permanent Grassland and Sown Ley: A Case Study in a Hemi-Boreal Region
Source: Plants (Basel). 2026 Oct 2;15(19):3026. doi: 10.3390/plants15193026 (PMC13667139; doi:10.3390/plants15193026)
Supplement: Supplementary file 1 [file plants-15-03026-s001.zip › Supplementary_File_S2_Biomass Yield, Chemistry, Composition, Functional diversity_Tables_S2-S7.pdf]

Supplementary Analyses of Biomass Yield, Chemistry, Botanical Composition, and Functional Diversity

This supplementary file provides detailed statistical results and diagnostics for biomass yield, biomass chemical composition, botanical-group composition, and plant functional diversity. It includes outcome-specific tests for fresh-matter yield (FMY), dry-matter yield (DMY), crude protein (CP), crude fat (CF), acid detergent fibre (ADF), neutral detergent fibre (NDF), total phenolic content (TPC), and DPPH radical-scavenging activity, together with unadjusted p-values, false discovery rate-adjusted q-values, and effect-size estimates. It also reports PERMANOVA and PERMDISP results for botanical-group composition. The functional-diversity component documents trait coverage, the species-pair Gower dissimilarity structure, plot-level functional-diversity diagnostics, the reduced-space quality of functional richness (FRic), and the sensitivity of Rao's quadratic entropy (RaoQ) and functional dispersion (FDis) to alternative Euclidean corrections.

The biomass yield, chemistry, and botanical-composition analyses quantify differences among the sampled quadrats within the two individual fields. Because the fields were not independently replicated at the grassland-system level, these results should be interpreted as field-specific comparisons rather than as independently replicated tests of ley and permanent-grassland systems. The analysed functional traits were life form, log-transformed seed mass, predominant dispersal mode, and dispersal-distance class. These traits represent dimensions of persistence, regeneration, and dispersal strategy. They are not direct measures of resource acquisition, forage digestibility, tissue nutrient composition, or secondary metabolism. Functional-diversity results are therefore interpreted with respect to community assembly and ecological-strategy structure and not as a mechanistic explanation of forage chemistry or beef lipid composition.

Table S2. Coverage of the four-trait matrix across the recorded vegetation.

Metric	Value
Taxa in vegetation matrix	90
Taxa with complete data for all four traits	88
Taxa matched	97.78%
Cumulative vegetation cover represented	98.68%
Cumulative cover represented in ley plots	100.00%
Cumulative cover represented in permanent grassland plots	97.84%

Note: The two excluded taxa were Equisetum arvense and E. palustre. Together, they represented 1.32% of total cumulative vegetation cover.

Table S3. Summary of the species-pair Gower dissimilarity matrix.

Statistic	Value
Number of taxa	88
Number of unique species pairs	3828
Minimum	0.000
First quartile (Q1)	0.046
Median	0.283
Mean	0.301
Third quartile (Q3)	0.487
Maximum	1.000
Standard deviation	0.233
Zero-distance species pairs	34
Zero-distance pairs	0.89%

Note: Life form and predominant dispersal mode were treated as nominal factors, log-transformed seed mass as a continuous variable, and dispersal-distance class as an ordered factor using the Podani treatment. All four traits received equal weight. Zero dissimilarity indicates identical profiles across the four analysed traits.

Table S4. Reduced-space and convex-hull diagnostics for plot-level FRic.

Diagnostic	Result
Positive PCoA axes in the corrected matrix	67
PCoA axes removed for FRic dimensionality reduction	64
PCoA axes retained for plot-level FRic	3
FRic reduced-space quality, qual.FRic	0.284
Minimum to maximum plot-level taxon richness	5 to 32
Minimum to maximum functionally singular taxa per plot	4 to 29
Plots with insufficient functionally singular taxa	0
Plots with missing FRic	0
Plots with zero FRic	0

Note: The corrected Gower matrix was not Euclidean before correction. Using the maximum dimensionality permitted by the minimum number of functionally singular taxa per plot (m = "max"), dbFD retained three PCoA axes because the minimum number of functionally singular taxa in a plot was four. Although all plots met the geometric requirement and yielded finite FRic values, the reduced-space quality was low. FRic was therefore excluded from the primary inferential comparison and retained only as exploratory supplementary information.

Table S5a. Sensitivity of the permanent grassland minus ley functional-diversity contrasts to the Euclidean correction.

Metric	Correction	Permanent grassland minus ley	Two-sided permutation p-value
RaoQ	Cailliez	0.0150	0.0002
RaoQ	Lingoes	0.0150	0.0002
FDis	Cailliez	0.0583	0.0026
FDis	Lingoes	0.0583	0.0026

Note: The square-root correction was evaluated but was not computationally applicable to the Gower distance matrix. Cailliez and Lingoes corrections were successfully applied and produced identical RaoQ and FDis contrasts and permutation p-values. The direction and magnitude of both contrasts were therefore consistent across the successfully applied correction methods.

The correction-sensitivity results indicate that the observed differences in RaoQ and FDis were not attributable to the choice between Cailliez and Lingoes corrections. However, these indices remain limited to the persistence, regeneration, and dispersal dimensions represented by the four-trait set. The functional-diversity and biomass-chemistry contrasts should therefore be interpreted as separate comparisons between the same two studied fields.

Table S5b. Plot-level functional-diversity metrics in the studied ley and permanent grassland fields.

Plot ID	Grassland	Species richness	Functionally singular species	FRic	RaoQ	FDis	FEve
L1	Ley	9	8	0.108127	0.017137	0.063508	0.479179
L2	Ley	7	6	0.012844	0.003041	0.023157	0.195951
L3	Ley	6	5	0.001972	0.003644	0.027458	0.530269
L4	Ley	8	7	0.019647	0.003530	0.024946	0.435075
L5	Ley	6	5	0.002537	0.016510	0.118368	0.442113
L6	Ley	6	5	0.000657	0.019507	0.139255	0.470009
L7	Ley	7	6	0.008776	0.003113	0.022560	0.497599
L8	Ley	10	9	0.125236	0.023064	0.084907	0.557681
L9	Ley	5	4	0.000077	0.000037	0.003411	0.666667
L10	Ley	6	5	0.000207	0.000106	0.007608	0.659092
L11	Ley	6	5	0.000293	0.018946	0.133498	0.638544
L12	Ley	5	4	0.000091	0.018699	0.134021	0.312020
L13	Ley	6	5	0.002537	0.007317	0.051031	0.750000

L14	Ley	6	5	0.000207	0.000087	0.006668	0.603602
L15	Ley	6	5	0.000207	0.000071	0.006535	0.750000
L16	Ley	5	4	0.000077	0.000035	0.003495	0.535447
P1	Permanent grassland	22	19	0.039358	0.014658	0.104006	0.586738
P2	Permanent grassland	20	17	0.187735	0.031170	0.171089	0.615048
P3	Permanent grassland	19	16	0.375214	0.017359	0.081509	0.576095
P4	Permanent grassland	27	23	0.632220	0.009122	0.041334	0.449087
P5	Permanent grassland	30	25	0.609016	0.028970	0.120696	0.624260
P6	Permanent grassland	32	29	0.815885	0.040232	0.167480	0.563250
P7	Permanent grassland	29	23	0.565246	0.025902	0.121488	0.558451
P8	Permanent grassland	14	12	0.102406	0.025300	0.094537	0.484627
P9	Permanent grassland	17	14	0.223976	0.019672	0.077536	0.662649
P10	Permanent grassland	23	20	0.159581	0.029746	0.165942	0.718609
P11	Permanent grassland	22	19	0.044706	0.016666	0.103238	0.638421
P12	Permanent grassland	30	25	0.609016	0.025258	0.114119	0.588396
P13	Permanent grassland	29	24	0.614552	0.020362	0.085893	0.605346

Note: Functional-diversity metrics were calculated separately for each 1 m^2 vegetation plot. RaoQ, FDis, and FEve were weighted by relative plant cover, whereas FRic was calculated without abundance weighting. None of the 29 plots had FDis equal to zero. Five of the 16 ley plots had FDis below 0.01, whereas none of the 13 permanent grassland plots had FDis below 0.01. FRic is provided for completeness but was excluded from the primary inferential comparison and treated as exploratory because of the low quality of the reduced-space representation (qual.FRic = 0.284). RaoQ, Rao's quadratic entropy; FDis, functional dispersion; FEve, functional evenness; FRic, functional richness.

Table S6. Biomass yield and chemical characteristics of the studied ley and permanent grassland fields.

**Outcome**	**Unit**	**Ley, mean ± SD (n = 16)**	**Permanent grassland, mean ± SD (n = 13)**	**Test**	**Statistic**	**Unadjusted p**	**FDR q**	**Effect size**
FMY	t ha^{-1}	12.91 ± 7.01	6.68 ± 4.10	Wilcoxon rank-sum	W = 150.0	0.0460	0.0613	Cliff's δ = 0.442
DMY	t ha^{-1}	2.92 ± 1.56	1.45 ± 0.86	Wilcoxon rank-sum	W = 163.0	0.0103	0.0165	Cliff's δ = 0.567
CP	% DM	14.85 ± 1.12	15.84 ± 3.06	Welch t-test	t = −1.105	0.2871	0.3281	Hedges' g = −0.435
CF	% DM	1.57 ± 0.27	1.55 ± 0.55	Welch t-test	t = 0.174	0.8637	0.8637	Hedges' g = 0.068
ADF	% DM	29.40 ± 3.97	34.91 ± 5.46	Wilcoxon rank-sum	W = 35.5	0.00285	0.00569	Cliff's δ = −0.659

NDF	% DM	47.60 ± 6.08	57.94 ± 7.94	Wilcoxon rank-sum	W = 33.5	0.00214	0.00569	Cliff's δ = −0.678
TPC	mg GAE g^{-1} DW	13.58 ± 3.65	22.33 ± 8.13	Welch t-test	t = −3.598	0.00243	0.00569	Hedges' g = −1.403
DPPH	µmol TE g^{-1} DW	57.19 ± 11.01	141.70 ± 63.20	Welch t-test	t = −4.763	0.000404	0.00323	Hedges' g = −1.914

Note. FMY, fresh-matter yield; DMY, dry-matter yield; CP, crude protein; CF, crude fat; ADF, acid detergent fibre; NDF, neutral detergent fibre; TPC, total phenolic content; GAE, gallic acid equivalents; TE, Trolox equivalents; DM, dry matter; DW, dry weight. Effect-size signs follow Ley minus permanent grassland. Benjamini–Hochberg correction was applied across the eight prespecified yield and chemistry outcomes. Welch's t-test was used for parametric two-group comparisons when approximate normality was adequately supported in both fields; Wilcoxon rank-sum tests were used when this condition was not met in at least one field. Individual quadrat-level observations are available in the MIDAS repository: https://doi.org/10.18279/MIDAS.560532

Table S7. Botanical-group composition of the studied ley and permanent grassland fields.

**Botanical group**	**Unit**	**Ley, mean ± SD (n = 16)**	**Permanent grassland, mean ± SD (n = 13)**
Legumes	% of within-quadrat botanical biomass	30.86 ± 14.90	7.11 ± 4.79
Grasses	% of within-quadrat botanical biomass	68.56 ± 15.69	40.38 ± 16.21
Forbs	% of within-quadrat botanical biomass	0.59 ± 1.63	46.73 ± 19.38
Sedges	% of within-quadrat botanical biomass	0.00 ± 0.00	5.79 ± 12.59

Note. Percentages were calculated separately within each quadrat and are reported as arithmetic means across quadrats. Consequently, they may differ from percentages calculated as ratios of field-level mean component yield to mean total yield. The four botanical-group proportions were evaluated jointly using one-way PERMANOVA based on Euclidean distances calculated from Hellinger-transformed within-quadrat proportions, with 9,999 permutations (pseudo-F = 64.36, $R^2 = 0.704$, $p < 0.001$). PERMDISP, also based on 9,999 permutations, indicated unequal multivariate dispersion between fields ($F = 5.03$, $p = 0.032$), with greater dispersion in the permanent grassland than in the ley (mean distance to centroid: 0.262 versus 0.164). Therefore, the PERMANOVA result may partly reflect unequal within-field heterogeneity in addition to differences in botanical-group composition. Separate univariate tests were not performed because the four proportions are interdependent and sum to 100%. Individual quadrat-level observations are available in the MIDAS repository: https://doi.org/10.18279/MIDAS.560532
