# Supplementary material for: Comparing Vegetation Characteristics and Production Outcomes of Semi-Natural Permanent Grassland and Sown Ley: A Case Study in a Hemi-Boreal Region
Source: Plants (Basel). 2026 Oct 2;15(19):3026. doi: 10.3390/plants15193026 (PMC13667139; doi:10.3390/plants15193026)
Supplement: Supplementary file 1 [file plants-15-03026-s001.zip › Supplementary_File_S3_Intramuscular fatty acid proportions_Table_S8.pdf]

Supplementary individual intramuscular fatty acid proportions in young bulls according to grassland group and year

Table S8. Individual intramuscular fatty acid proportions in young bulls according to grassland group and year. Values are percentages of total identified fatty acids and are presented as arithmetic mean ± standard deviation.

Fatty acid	Ley 2019 (n = 4)	Permanent grassland 2019 (n = 4)	Ley 2020 (n = 4)	Permanent grassland 2020 (n = 4)
**C6:0**	0.053 ± 0.025	0.092 ± 0.109	0.019 ± 0.006	0.028 ± 0.011
**C8:0**	0.070 ± 0.040	0.048 ± 0.024	0.053 ± 0.038	0.048 ± 0.012
**C10:0**	0.054 ± 0.027	0.044 ± 0.027	0.458 ± 0.243	0.223 ± 0.168
**C11:0**	0.030 ± 0.022	0.013 ± 0.009	0.016 ± 0.003	0.022 ± 0.010
**C12:0**	0.325 ± 0.035	0.396 ± 0.074	0.405 ± 0.056	0.415 ± 0.186
**C13:0**	0.117 ± 0.049	0.126 ± 0.088	0.105 ± 0.048	0.091 ± 0.058
**C14:0**	3.825 ± 0.371	2.785 ± 0.429	5.466 ± 1.107	4.968 ± 1.220
**C15:0**	1.413 ± 0.519	1.486 ± 0.633	1.453 ± 0.620	1.073 ± 0.922
**C16:0**	23.299 ± 0.748	20.952 ± 0.714	19.996 ± 1.684	18.956 ± 0.243
**C17:0**	1.353 ± 0.181	1.426 ± 0.235	2.341 ± 0.411	2.147 ± 0.296
**C18:0**	19.204 ± 0.575	16.204 ± 0.503	17.948 ± 0.904	14.950 ± 1.705
**C20:0**	0.820 ± 0.080	0.547 ± 0.105	0.399 ± 0.157	0.336 ± 0.151
**C21:0**	0.138 ± 0.010	0.144 ± 0.021	0.115 ± 0.051	0.242 ± 0.197
**C22:0**	0.230 ± 0.008	0.221 ± 0.041	0.175 ± 0.072	0.173 ± 0.063
**C23:0**	0.162 ± 0.113	0.383 ± 0.140	0.096 ± 0.047	0.157 ± 0.088
**C24:0**	0.114 ± 0.023	0.091 ± 0.046	0.138 ± 0.018	0.145 ± 0.013
**C14:1**	0.926 ± 0.309	1.562 ± 0.815	1.337 ± 0.528	1.001 ± 0.917
**C15:1 cis-10**	0.558 ± 0.324	0.647 ± 0.536	0.245 ± 0.066	0.242 ± 0.112
**C16:1 cis-9**	4.133 ± 0.321	4.265 ± 1.989	5.041 ± 0.299	5.896 ± 0.820
**C17:1 cis-10**	1.416 ± 0.313	1.444 ± 0.388	1.702 ± 0.718	1.238 ± 0.896
**C18:1 cis-9**	25.838 ± 0.946	28.934 ± 1.594	27.537 ± 0.615	29.960 ± 0.457
**C20:1 cis-11**	0.377 ± 0.199	0.581 ± 0.221	0.489 ± 0.108	0.657 ± 0.208
**C22:1 cis-13**	0.185 ± 0.120	0.195 ± 0.192	0.248 ± 0.090	0.242 ± 0.061
**C18:2 n-6**	4.508 ± 0.285	4.476 ± 0.700	2.790 ± 0.122	4.303 ± 1.167
**C18:3 n-6**	2.611 ± 0.517	3.530 ± 0.892	1.104 ± 0.030	1.638 ± 0.611
**C18:3 n-3**	2.839 ± 0.718	3.631 ± 0.243	0.231 ± 0.043	1.990 ± 0.389
**C20:2 cis-11,14**	0.237 ± 0.111	0.951 ± 1.131	0.394 ± 0.130	0.679 ± 0.189
**C20:3 n-6**	0.951 ± 0.584	0.846 ± 0.424	0.328 ± 0.091	0.380 ± 0.219

**C20:3 n-3**	0.521 ± 0.588	0.524 ± 0.530	0.299 ± 0.146	0.383 ± 0.190
**C20:4 n-6**	0.118 ± 0.084	0.085 ± 0.002	0.572 ± 0.298	0.741 ± 0.278
**C20:5 n-3 (EPA)**	0.631 ± 0.089	0.711 ± 0.174	0.234 ± 0.079	0.466 ± 0.446
**C22:2 cis-13,16**	0.206 ± 0.213	0.109 ± 0.151	0.374 ± 0.137	0.211 ± 0.090
**C22:6 n-3 (DHA)**	0.137 ± 0.011	0.123 ± 0.031	0.655 ± 0.936	0.252 ± 0.239
**Unresolved trans-C18:1 fatty acids**	2.375 ± 0.494	2.214 ± 0.282	5.040 ± 1.699	2.396 ± 0.295
**trans-C18:2 fatty acids**	0.224 ± 0.296	0.213 ± 0.282	2.204 ± 0.849	3.352 ± 0.196
**C18:1 trans-11 (vaccenic acid)**	Not resolved or quantified separately	Not resolved or quantified separately	Not resolved or quantified separately	Not resolved or quantified separately
**CLA cis-9,trans-11**	Separate identification not verifiable	Separate identification not verifiable	Separate identification not verifiable	Separate identification not verifiable
**C22:5 n-3 (DPA)**	Not identified or quantified separately	Not identified or quantified separately	Not identified or quantified separately	Not identified or quantified separately

Note: Values are arithmetic means ± standard deviations and are expressed as percentages of total identified fatty acids. Each grassland group × year combination included four animals (n = 4). Fatty acid methyl esters were identified by comparing sample retention times with those of the Supelco 37 Component FAME Mix (Catalog No. 47885-U). No additional reference standards for individual C18:1 trans positional isomers were used. Although the reference mixture contained methyl elaidate (C18:1 trans-9), it did not contain vaccenic acid (C18:1 trans-11) or the range of other C18:1 trans positional isomers required to establish the identity of the trans-C18:1 region. Because the original chromatograms and detailed retention-time records were unavailable for retrospective verification, the component previously assigned specifically to elaidic acid is conservatively reported as unresolved trans-C18:1 fatty acids. Vaccenic acid was not quantified separately. Separate identification of CLA cis-9,trans-11 could not be verified from the retained analytical records.
