# Supplementary material for: Comparing Vegetation Characteristics and Production Outcomes of Semi-Natural Permanent Grassland and Sown Ley: A Case Study in a Hemi-Boreal Region
Source: Plants (Basel). 2026 Oct 2;15(19):3026. doi: 10.3390/plants15193026 (PMC13667139; doi:10.3390/plants15193026)
Supplement: Supplementary file 1 [file plants-15-03026-s001.zip › Supplementary_File_S4_IMF Sensitivity Analysis_Table_S9.pdf]

Supplementary sensitivity analysis

Fatty-acid models were refitted with individual intramuscular fat content included as a covariate. The table compares the original and IMF-adjusted grassland-group contrasts. In accordance with the revised fatty-acid classification, MUFA is reported as cis-MUFA because trans fatty acids were assigned to a separate trans FA class. The trans FA result is considered exploratory because individual trans-C18:1 positional isomers could not be reliably assigned.

Table S 9. Sensitivity analysis of relative intramuscular fatty acid composition, fatty acid ratios, and calculated lipid-quality indices after adjustment for individual intramuscular fat content.

Outcome	Base group contrast	95% CI	IMF-adjusted group contrast	95% CI	IMF coefficient	95% CI	Group q-value after IMF adjustment
SFA	-0.230	-0.266 to -0.194	-0.233	-0.268 to -0.198	-0.022	-0.059 to 0.015	<0.001
cis-MUFA	0.147	0.061 to 0.233	0.147	0.055 to 0.238	-0.001	-0.097 to 0.094	0.008
PUFA	0.332	0.121 to 0.543	0.342	0.122 to 0.561	0.074	-0.156 to 0.305	0.008
trans FA	-0.155	-0.345 to 0.035	-0.146	-0.344 to 0.051	0.067	-0.140 to 0.274	0.130
n-3 PUFA	0.530	0.155 to 0.904	0.565	0.203 to 0.928	0.268	-0.112 to 0.649	0.008
n-6 PUFA	0.257	0.043 to 0.471	0.258	0.031 to 0.485	0.005	-0.233 to 0.243	0.032
PUFA/SFA	0.097	0.045 to 0.150	0.099	0.043 to 0.154	0.009	-0.049 to 0.067	0.002
log (n-6/n-3)	-0.280	-0.645 to 0.085	-0.314	-0.669 to 0.041	-0.258	-0.630 to 0.114	0.084
AI	-0.206	-0.278 to -0.134	-0.213	-0.282 to -0.144	-0.056	-0.128 to 0.017	<0.001
TI	-0.439	-0.538 to -0.340	-0.447	-0.546 to -0.348	-0.059	-0.163 to 0.045	<0.001
h/H	0.397	0.271 to 0.523	0.400	0.267 to 0.532	0.018	-0.121 to 0.158	<0.001

Note. Group contrasts are expressed as permanent grassland minus ley. SFA, cis-MUFA, PUFA, trans FA, n-3 PUFA, and n-6 PUFA were analysed as proportions using the logit transformation. The n-6/n-3 ratio was analysed after natural-log transformation, whereas PUFA/SFA, AI, TI, and h/H were analysed on their original scales. Accordingly, the group contrasts, 95% confidence intervals, and IMF coefficients are reported on the respective model scales. IMF coefficients represent the estimated change in the model outcome associated with a one-percentage-point increase in intramuscular fat content, after adjustment for studied field and year. The reported q-values are Benjamini–Hochberg-adjusted Freedman–Lane permutation p-values for the studied-field contrast in the IMF-adjusted models. The trans FA result was considered exploratory because of uncertainty in the identification of individual trans positional isomers. Abbreviations: AI, atherogenicity index; CI, confidence interval; cis-MUFA, cis-monounsaturated fatty acids; h/H, hypocholesterolemic-to-hypercholesterolemic fatty acid ratio; IMF, intramuscular fat; PUFA, polyunsaturated fatty acids; SFA, saturated fatty acids; TI, thrombogenicity index.
