# Supplementary material for: Comparing Vegetation Characteristics and Production Outcomes of Semi-Natural Permanent Grassland and Sown Ley: A Case Study in a Hemi-Boreal Region
Source: Plants (Basel). 2026 Oct 2;15(19):3026. doi: 10.3390/plants15193026 (PMC13667139; doi:10.3390/plants15193026)
Supplement: Supplementary file 1 [file plants-15-03026-s001.zip › Supplementary_File_S5_Study-Area Mapping Sampling Design_Figure_S3_Table_S10.pdf]

1. Supplementary Methods

1.1. Study-area mapping

The study-area map was prepared in R. Grazing-field boundaries, woody-vegetation patches and sampling locations were processed in the Lithuanian Coordinate System LKS-94 / Lithuania TM (EPSG:3346). Field boundaries and woody vegetation patches were digitised by the authors using the 2018 Lithuanian digital raster orthophoto map ORT10LT as the spatial reference at a nominal scale of 1:10,000 [47]. Lithuanian administrative boundaries and the boundary of Kėdainiai District Municipality were obtained from the Lithuanian INSPIRE Administrative Units dataset [48]. The European locator map used Natural Earth country boundaries. The detailed map was prepared from the original spatial layers and exported at a final width suitable for a two-column journal page.

1.2. Sampling plots and coordinates

Vegetation composition and above-ground biomass were sampled from the same 1 m × 1 m quadrats. In total, 29 quadrats were included: 13 in the permanent grassland and 16 in the ley. Plot codes beginning with P identify permanent-grassland quadrats, whereas codes beginning with L identify ley quadrats. WGS84 coordinates were obtained from the sampling-point spatial layer and are reported in decimal degrees in Table S1. The same plot codes are used in the vegetation, functional-diversity and biomass datasets.

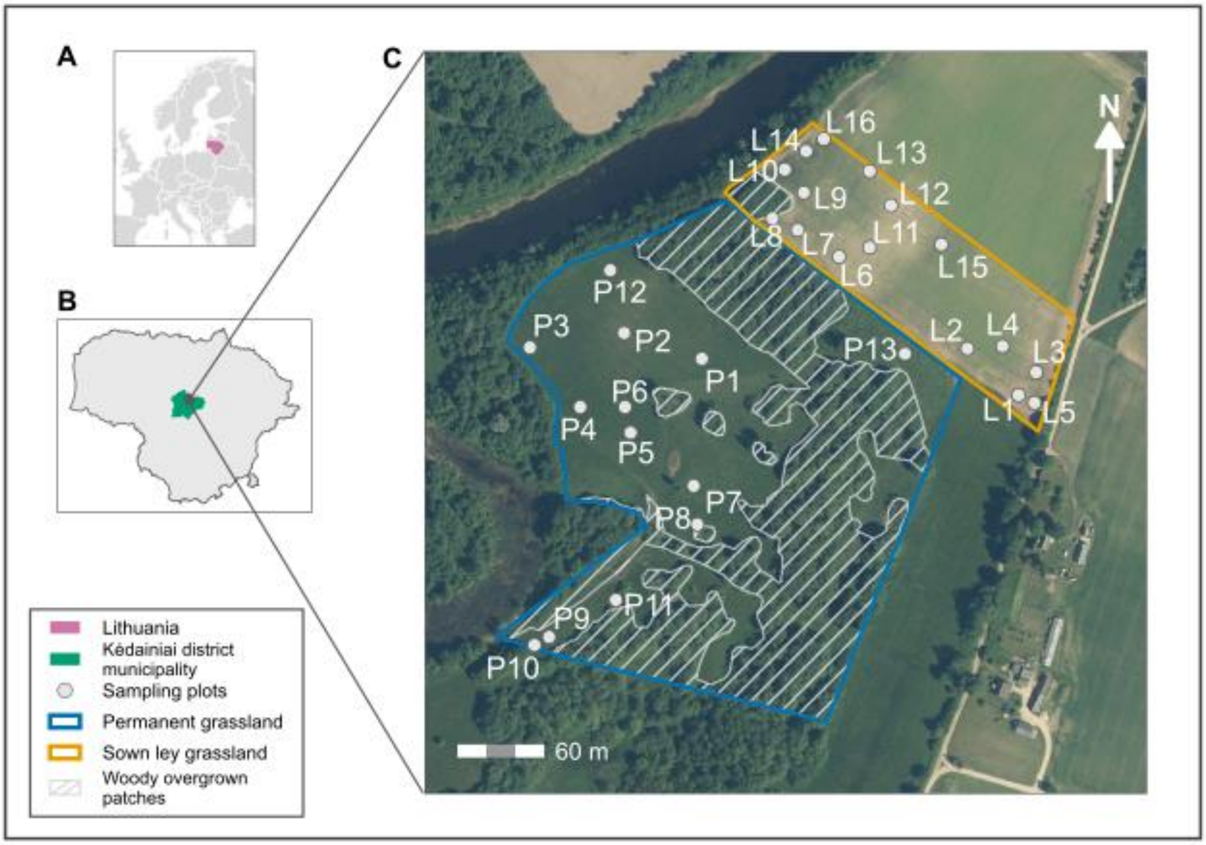

Figure S3. Location of the study area and sampling design in the Nevėžis River valley, central Lithuania. (A) Location of Lithuania within Europe; (B) location of Kėdainiai District Municipality and the study site; and (C) boundaries of the permanent grassland and ley, woody-vegetation patches, and vegetation and biomass sampling plots. The detailed map is

shown over the ORT10LT 2018 orthophotographic background [47]; administrative boundaries were obtained from the Lithuanian INSPIRE Administrative Units dataset [48].

Table S10. Codes, grassland assignments and WGS84 coordinates of the vegetation and biomass sampling plots shown in Figure S3.

Plot code	Grassland type	Longitude (WGS84)	Latitude (WGS84)
P1	Permanent grassland	24.022050	55.383060
P2	Permanent grassland	24.021200	55.383220
P3	Permanent grassland	24.020170	55.383130
P4	Permanent grassland	24.020720	55.382760
P5	Permanent grassland	24.021270	55.382600
P6	Permanent grassland	24.021210	55.382760
P7	Permanent grassland	24.021960	55.382270
P8	Permanent grassland	24.022000	55.382030
P9	Permanent grassland	24.020380	55.381330
P10	Permanent grassland	24.020220	55.381280
P11	Permanent grassland	24.021110	55.381560
P12	Permanent grassland	24.021050	55.383610
P13	Permanent grassland	24.024270	55.383090
L1	Ley	24.025511	55.382833
L2	Ley	24.024952	55.383123
L3	Ley	24.025703	55.382975
L4	Ley	24.025336	55.383137
L5	Ley	24.025686	55.382785
L6	Ley	24.023549	55.383694
L7	Ley	24.023098	55.383860
L8	Ley	24.022823	55.383932
L9	Ley	24.023165	55.384093
L10	Ley	24.022957	55.384236
L11	Ley	24.023883	55.383751
L12	Ley	24.024117	55.384012
L13	Ley	24.023884	55.384226
L14	Ley	24.023190	55.384350
L15	Ley	24.024668	55.383770
L16	Ley	24.023383	55.384426

Note: P, permanent grassland; L, ley. Coordinates are reported in decimal degrees using WGS84.
