# Supplementary material for: Comparing Vegetation Characteristics and Production Outcomes of Semi-Natural Permanent Grassland and Sown Ley: A Case Study in a Hemi-Boreal Region
Source: Plants (Basel). 2026 Oct 2;15(19):3026. doi: 10.3390/plants15193026 (PMC13667139; doi:10.3390/plants15193026)
Supplement: Supplementary file 1 [file plants-15-03026-s001.zip › Supplementary_File_S6_PlntBERT-Based EUNIS Classification_Figure_S4_Table_S11.pdf]

1. Supplementary Methods

1.1. Scope and interpretation

Vegetation classification was performed separately for individual 1 × 1 m microplots sampled in the studied ley and permanent-grassland fields. Pl@ntBERT was trained predominantly on vegetation-plot data from the European Vegetation Archive (EVA) [50,51], whereas the present records were obtained from 1 m^2 microplots, which are smaller than the grassland plots typically represented in the training data and the sampling units normally used for formal habitat mapping. This domain mismatch in plot size was the primary reason for interpreting the predictions only as descriptive indicators of fine-scale floristic affinity and classification consistency, rather than as an independent habitat determination, validation, or reclassification of Annex I habitat status. Field-level classification consistency was summarised using the modal Top-1 EUNIS assignment among microplots within each field.

1.2. Data preprocessing and taxonomic harmonisation

The vegetation matrix contained plot identifiers, grassland assignments and taxon-level Braun–Blanquet cover values. Species-cover columns were converted to numeric values, missing values were replaced by zero and the matrix was converted to long format. Selected taxon names were harmonised before model input to improve compatibility with the nomenclatural concepts represented in European vegetation datasets. The substitutions applied were: *Elytrigia repens* to *Elymus repens*; *Cerastium holosteoides* to *Cerastium fontanum*; *Festulolium* to × *Festulolium*; *Alchemilla vulgaris* to *Alchemilla vulgaris* group; *Taraxacum officinale* to *Taraxacum officinale* group; and *Rhamnus catharticus* to *Rhamnus cathartica*. Plant nomenclature was checked against Euro+Med PlantBase [53].

1.3. Pl@ntBERT input and classification

For each microplot, taxa with zero cover were removed, and the remaining taxa were ordered by decreasing cover. The ordered taxon names were concatenated into a single text sequence representing one microplot. EUNIS classification was performed using the official Pl@ntBERT text-classification model, CesarLeblanc/plantbert_text_classification_model (Hugging Face Hub, Git commit ef1812f), and the authors' Python-based inference pipeline [50]. The workflow was executed in Google Colab. For each microplot, the three highest-ranked level-3 EUNIS predictions were requested using --k_habitat 3, and the Top-1, Top-2, and Top-3 classes were retained. The pipeline output retained the ranked classes but did not report their prediction confidence scores. The EUNIS habitat classification was used as the reference framework [52].

Classification consistency was summarised separately for the two fields as the proportion of microplots assigned to the modal Top-1 EUNIS code. The consistency value was calculated as the number of microplots assigned to the modal code divided by the total number of microplots within the field. This was treated as a descriptive index of fine-scale floristic consistency, not as a formal estimate of classification accuracy.

2. Supplementary Results

All 13 permanent-grassland microplots were assigned to EUNIS class R22 as the Top-1 prediction, corresponding to modal-code consistency of 100%. In the ley, V38 was the modal Top-1 prediction and occurred in 8 of 16 microplots, corresponding to 50% consistency. The remaining ley microplots were assigned to R21, V39, V11, R22 or U37. Thus, the permanent grassland showed

a coherent fine-scale floristic signal, whereas the ley showed a more heterogeneous pattern of EUNIS affinities (Table S11; Figure S4).

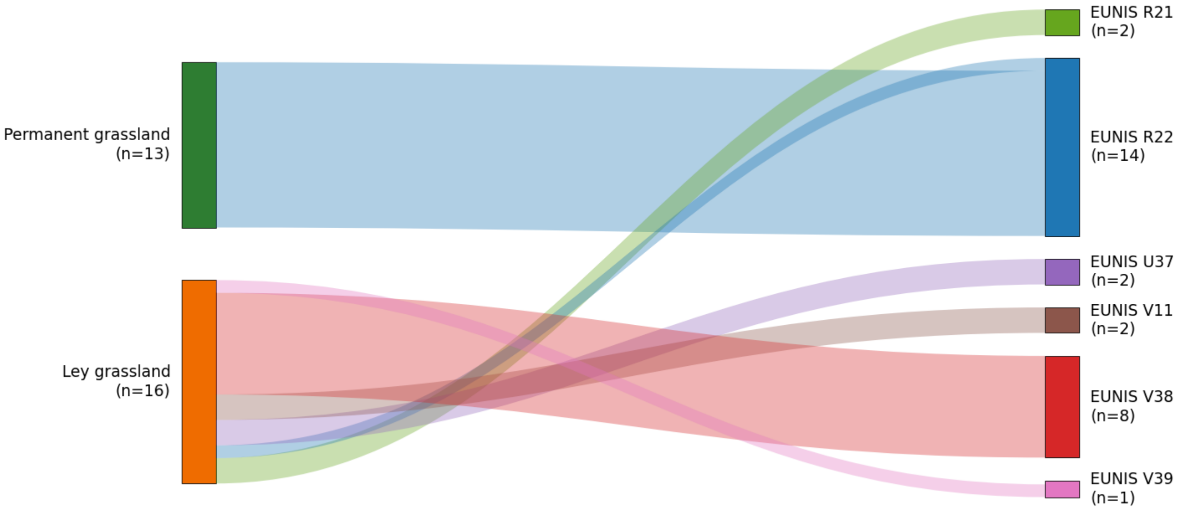

Figure S4. Flow of individual 1 m × 1 m microplots from grassland field to the Top-1 EUNIS class predicted by Pl@ntBERT. Band width represents the number of microplots. The figure illustrates the consistent R22 assignment of the permanent-grassland microplots and the more heterogeneous classification of the ley microplots.

Table S11. Summary of Pl@ntBERT Top-1 EUNIS classification consistency for the two studied fields.

Field type	Microplots	Modal Top-1 EUNIS	Modal frequency	Consistency	Other Top-1 codes
Permanent	13	R22	13 of 13	100.0%	None
Ley	16	V38	8 of 16	50.0%	R21, V39, V11, R22, U37

Note: The consistency value is the proportion of microplots assigned to the modal Top-1 EUNIS code within each field. It is a descriptive measure and not a formal accuracy estimate.
